# Narrative medicine intervention for mental wellbeing in Juvenile Myositis and juvenile idiopathic arthritis

**DOI:** 10.1186/s12969-026-01232-5

**Published:** 2026-05-31

**Authors:** Aviya L. Levy, Emily Steelquist, Aryn Bartley, Claire Unis, Courtney Wells, Elizabeth Dorn, Natalie Rosenwasser, Todd Edwards, Christian Lood, Susan Shenoi

**Affiliations:** 1 Rady Children’s Hospital, San Diego, CA USA; 2 Oregon Health & Science University, Portland, OR USA; 3 Lane Community College, Eugene, OR USA; 4 Sutter Health, San Francisco, CA USA; 5 University of Wisconsin River Falls, River Falls, WI USA; 6 University of Washington, Seattle, WA USA; 7 Pediatric Rheumatology, Division of Allergy, Immunology and Rheumatology, Department of Pediatrics, Rady Children’s Hospital, 3020 Children’s Way, San Diego, CA 92123 USA; 8 Seattle Children’s Hospital and Research Center, University of Washington, Seattle, WA USA

**Keywords:** Narrative medicine, Juvenile idiopathic arthritis, Juvenile dermatomyositis, Mental health

## Abstract

**Background:**

Children with juvenile dermatomyositis (JDM) and juvenile idiopathic arthritis (JIA) have increased rates of anxiety and depression (15–65%) when compared to healthy children. Narrative medicine, a group-based intervention that allows patients to reflect on medical experiences, improves patient-reported outcomes with reduces depression rates in adults. Given limited data in pediatric rheumatology, this study assesses feasibility of a patient-targeted narrative medicine intervention and its impact on mental health burden in JDM and JIA.

**Methods:**

We prospectively recruited patients ages 6 to 21 with JDM and JIA for a narrative medicine intervention. Participants were divided by diagnosis and age into four narrative medicine groups, with six sessions held over 3 months. Demographic and medical information were collected by chart review. Patients completed pre- and post-intervention questionnaires including Patient-Reported Outcomes Measurement Information System Depression Scale, Generalized Anxiety Disorder-7 (GAD-7), Childhood Attitude Towards Illness Scale, Patient Health Questionnaire-8 (PHQ-8), and CoVID Stress Scale.

**Results:**

Twelve patients with JDM (67% female) and nine with JIA (78% female) participated in the narrative medicine intervention. All patients participated in at least 50% of sessions and 91% participated in at least four of the six sessions. Wilcoxon signed-rank test revealed no statistically significant difference between pre- and post-intervention questionnaires for any scales. Sub-analysis of those with elevated GAD-7 and PHQ-8 pre-intervention showed a trend towards improvement in anxiety (p=0.057) and no change for depression (p=0.171).

**Conclusion:**

Ninety-one percent participated in at least four of the six sessions, demonstrating feasibility. This exploratory study showed trend toward improved anxiety following narrative medicine session participation for those with elevated GAD-7.

**Supplementary Information:**

The online version contains supplementary material available at 10.1186/s12969-026-01232-5.

Juvenile Dermatomyositis (JDM) and Juvenile Idiopathic Arthritis (JIA) are two rare chronic autoimmune inflammatory diseases that affect children, with JDM affecting 2 to 4 in 1 million children and JIA affecting approximately 305 in 100,000 children [1, 2]. Children with JDM and JIA may have an impaired quality of life and increased rates of anxiety and depression, even during disease remission, with anxiety and depression rates ranging from 15 to 65% and suicidal ideation rates ranging from 14–34% [3-6]. The prevalence of mood disturbance among patients with JDM and JIA is disproportionately higher than prevalence among healthy children, and in some studies, these rates among patients with JDM and JIA are higher than those with special health care needs or other chronic diseases such as diabetes or asthma [3]. Furthermore, recent literature suggests that individuals with childhood-onset rheumatologic disease may be at higher risk for mental health disorders than individuals with adult-onset rheumatologic disease [6]. Research shows that mental illness for pediatric patients with chronic diseases can lead to poor outcomes during childhood and affect development and risk of mental illness in adulthood [3–6]. While early recognition and treatment of mental health disorders improves outcomes, there is a significant mental health care gap for youth with rheumatologic diseases. Workforce data from the American Academy of Child and Adolescent Psychiatry shows there are approximately 8,300 practicing child and adolescent psychiatrists in the US with over 15 million youth in need of this specialized care. Given this shortage and limited mental health provider availability with limited availability and accessibility of alternative interventions such as group cognitive behavioral therapy or art therapy, we must investigate alternative avenues to offer support for the aforementioned at-risk youth [3]. This study looked to investigate the feasibility of using narrative medicine as one such adjunct to standard of care medical treatment for children with rheumatic disease who are in need of mental health support.

Narrative medicine is a patient-targeted group-based intervention that allows patients to reconstruct their medical experiences through written or oral portrayals of emotions and self-reflective perspectives. It has demonstrated improved patient-reported outcomes with reduced rates of depression in adults [7]. Research suggests these group-sharing experiences improve quality of life and offer a therapeutic venue for patients with chronic diseases to support one another [8]. The mental health burden has dramatically increased during the current COVID-19 pandemic, reflecting an ongoing need for avenues to mitigate mental health burden [9]. Recognizing there is limited data to date investigating mental health improvements following narrative medicine interventions, this explorative study aimed to determine if patient-targeted narrative medicine interventions improve mental health burden for individuals with JDM and JIA.

There is limited data on use of narrative medicine interventions within the rheumatologic patient population. Our prior work shows feasibility of implementation of a storytelling intervention with patients with rheumatologic diseases. The published study involved one-hour video-based narrative medicine sessions that resulted in improvement in self-reported physical health for participants following completion of the study [10]. The current study aims to build upon this prior pilot data so as to implement narrative-based intervention on a broader scale and maximize access to care for individuals with higher risk of mental health burden.

## Materials and methods

### Study design

This primary outcome of interest was assessing the feasibility of implementing a narrative medicine intervention for patients with JDM and JIA. Participation rate and completion rate of pre- and post-participation questionnaires were monitored to assess feasibility. Data collected included baseline mental health burden in patients with JDM compared to patients with JIA, as well as pre- and post-participation patient-reported outcomes for patients with JDM and JIA. The narrative medicine intervention consisted of 3 months of bi-monthly 1-hour video-based narrative medicine sessions oriented around health-related interactions. Sessions were conducted in disease specific groups (JDM/JIA) of 4–6 patients per session, with two narrative medicine trained facilitators per session.

Eight narrative medicine facilitators were recruited through the Northwest Narrative Medicine Collaborative, an organization focused on training narrative medicine facilitators and sharing narrative medicine with the medical community. All facilitators underwent training prior to implementation of the curriculum, including introductory training on the disease course for JDM and JIA, as well as developmental expectations of participants. Moderators were assigned based on narrative medicine expertise and prior experience supporting varied participant age ranges. Facilitators were integrated into the curriculum editing process, and feedback was solicited from facilitators during and following completion of the narrative medicine sessions. Participants of all ages as well as parents of participants completed written feedback following completion of the intervention. Consistency and inter-rater agreement among moderators were assessed through routine patient and parent surveys as well as moderator check-ins that occurred after each session.

Age-appropriate interventions including poetry, photography, and art were used to engage participants and prompt discussions around unique medical experiences (See Appendix 1). Art supplies and writing materials were mailed to participants. A Seattle Children’s psychologist through the Seattle Children’s Rheumatology Clinic was made available for any patients as needed, and individuals with positive mental health screens were automatically referred for further care with the clinical psychologist, with treatment initiated following completion of the study period.

### Setting

Participants were recruited through Seattle Children’s Hospital (SCH) and the SCH Juvenile Myositis Clinic of Excellence. Enrolled patients participated in a bi-monthly 1-hour video-based narrative medicine session, for a total of six sessions. Sessions were held virtually to maximize accessibility.

## Ethics Review

Ethical approval was obtained through the SCH Institutional Review Board (STUDY00003804).

### Sampling

Patients with JIA and JDM were consented through convenience sampling of patients with JDM and JIA who were seen in clinic between June 2023 and September 2023. Inclusion criteria included patients aged 6 to 21 with a diagnosis of JDM and JIA who spoke English, with non-English speakers excluded from the study due to the nature of the narrative medicine intervention being led in English. Twenty dollars were provided to participants as a token of appreciation for survey completion.

### Participants

This longitudinal cohort study prospectively recruited patients with established diagnoses of JDM as well as age-matched patients with established diagnoses of JIA. Patients with JDM from 6 to 21 years of age, and patients with JIA from 6 to 21 years of age were included. These patients were identified through clinic appointments as well as through referrals by attending physicians at SCH. As the most prevalent rheumatologic condition seen in rheumatology clinic, JIA was selected over healthy patients as an age-matched control because narrative medicine interventions for patients to date have been targeted towards individuals living with chronic illness rather than healthy controls.

### Measures

Participants completed RedCap surveys on demographic and medical information and questionnaires. Disease activity was documented by clinicians in clinic visits prior to and following completion of the narrative medicine intervention. These disease activity measures included Physician-Reported Global scores for patients with JDM and JIA, where a score of 0 indicates no disease and a score of 10 indicates doing very poorly [11]. Disease activity for patients with JDM was also measured using the Manual Muscle Testing-8 (150 points indicates full function without muscle weakness), Childhood Myositis Assessment Scale (52 points indicates full function without muscle weakness), and Cutaneous Dermatomyositis Disease Area and Severity Index (100 activity points indicates significant skin activity, while 32 damage points indicates significant skin damage) [12–14]. Due to limited availability of patient scoring data for patients with JIA, Juvenile Arthritis Disease Activity Score was omitted. Depressive and anxiety symptoms were assigned using the Patient-Reported Outcomes Measurement Information System (PROMIS) Depression Scale, Patient Health Questionnaire-8 (PHQ-8) and General Anxiety Disorder-7 (GAD-7) questionnaires. For the PROMIS Depression Scale, which is validated for proxy-report ages 5 to 17 and for self-report ages 8–17, a T-score of 50 represents the average depression score for the United States general population, with a score above 50 representing worse than average and a score under 50 representing better than average [15]. For the PHQ-8, validated for ages 11 and up, a score of 0 to 4 points reflects normal or minimal depression, 5 to 9 points indicates mild depression, 10 to 14 points indicates moderate depression, and 15 or more points is concerning for severe depression [16]. For the GAD-7, validated for ages 11 and up, a score of 5 to 9 represents mild anxiety, a score of 10 to 14 represents moderate anxiety, and a score greater than 15 represents severe anxiety [17]. For the Childhood Attitude Toward Illness Scale (CATIS), scores range from 13 to 65 where a higher score indicates a more positive attitude towards the condition [18]. For the COVID Student Stress Questionnaire (CSSQ), scores range from 0 to 28 where a higher score suggests higher stress in the setting of the COVID-19 pandemic [19]. Of note, no validated measures were available for these measurements in the studied population. Total time between pre- and post-questionnaires was targeted at 120 days (pre-participation questionnaire to be completed within two weeks prior to intervention initiation and post-participation questionnaire to be completed within 2 weeks after intervention completion).

### Statistical Analysis

Descriptive analyses were used for patient demographics. Surveys completed pre- and post-intervention were assessed for differences with a Wilcoxon signed-rank test. Statistical analyses were conducted using RStudio 2023.09.1, Build 494 for Windows.

Given that this is a feasibility study, process measures included participation rate and ability to complete pre- and post-participation surveys. Feasibility criteria were established a priori, with feasible participation rate in at least 4 of the 6 sessions determined to be greater than or equal to 80% participation and feasible pre- and post-participation survey completion determined to be greater than or equal to 80%. Exploratory analysis was used to understand potential impact that may be of interest for future studies implementing narrative medicine interventions for a larger patient population.

## Results

### Study Participants

Twelve patients with JDM and nine age-matched patients with JIA participated in the narrative medicine sessions (Table 1). Narrative groups included one group of five patients with JIA and JDM ages 7–11, one group of five patients with JIA ages 12–19, one group of five patients with JDM ages 8–11, and one group of six patients with JDM ages 12–18. 91% of patients participated in 4 or more sessions, and 9% of patients participated in 3 sessions. Mean (SD) age of diagnosis (years) was 4.8 (2.5) and mean age of participation (years) was 12.4 (4.0). 50% of approached patients with JDM and 60% of approached patients with JIA consented to participate.

**Table 1:** Participant characteristics for patients with JDM and JIA

Characteristic	**JDM**	**JIA**	**All Participants**
	(n=12) N (%)	(n=9) N (%)	(n=21) N (%)
Self-reported Gender			
Female	8 (67)	7 (78)	15 (71)
Male	3 (25)	1 (11)	4 (19)
Prefer not to answer	1 (8)		1 (5)
No response		1(11)	1 (5)
Self-reported race+			
White	7 (58)	5 (56)	12 (57)
Native Hawaiian or Other Pacific	1 (8)	0 (0)	1 (5)
Islander			
More than one race	2 (16)	2 (22)	4 (19)
Unknown		1 (11)	1 (5)
Prefer not to answer	1 (8)		1 (5)
No response	1 (8)	1 (11)	2 (10)
Self-reported Ethnicity~			
Hispanic/Latino	3 (25)	1 (11)	4 (19)
Not Hispanic/Latino	8 (67)	6 (67)	14 (67)
Unknown		1 (11)	1 (5)
Prefer not to answer	1 (8)		1 (5)
No response		1 (11)	1 (5)
Other Labs			
ANA positive	N/A	6 (67)	N/A
ANA negative	N/A	3 (33)	N/A
HLA-B27 positive	N/A	0 (0)	N/A
HLA-B27 negative	N/A	8 (89)	N/A
HLA-B27 unknown	N/A	1 (11)	N/A
Rheumatoid Factor positive	N/A	0 (0)	N/A
Rheumatoid Factor negative	N/A	9 (100)	N/A
Anti-CCP positive	N/A	0 (0)	N/A
Anti-CCP negative	N/A	9 (100)	N/A
Myositis Antibody Panel*			
Negative	7 (58)	N/A	N/A
P155/140	2 (16)	N/A	N/A
Mi2	3 (25)	N/A	N/A
MJ	1 (8)	N/A	N/A
Disease-Related Complications			
Cataracts	1 (8)	0 (0)	1 (5)
Calcinosis	1 (8)	0 (0)	1 (5)
Pharyngeal weakness	1 (8)	0 (0)	1 (5)
Lipodystrophy	1 (8)	0 (0)	1 (5)
Boutonnieres deformity	0 (0)	1 (11)	1 (5)
Leg-length discrepancy	0 (0)	1 (11)	1 (5)
TNF-induced psoriasis	0 (0)	1 (11)	1 (5)
Comorbidities			
Asthma/Reactive Airway Disease	1 (8)	1 (11)	2 (10)
Congenital Hypothyroidism	1 (8)	0 (0)	1 (5)
Hypersensitivity vasculitis	0 (0)	1 (11)	1 (5)
Pain amplification	0 (0)	1 (11)	1 (5)
PCOS	0 (0)	1 (11)	1 (5)
Recurrent Urinary Tract Infections	1 (8)	0 (0)	1 (5)
Medication changes between surveys			
No change	9 (75)	2 (22)	11 (52)
Steroid wean	1 (8)	1 (11)	2 (10)
Plaquenil wean	1 (8)	0 (0)	1 (5)
Plaquenil started	0 (0)	1 (11)	1 (5)
Abatacept started	1 (8)	0 (0)	1 (5)
Adalimumab started	0 (0)	1 (11)	1 (5)
Etanercept started	0 (0)	1 (11)	1 (5)
Golimumab started	0 (0)	1 (11)	1 (5)
Leflunomide dose and frequency increased	0 (0)	1 (11)	1 (5)
Tocilizumab frequency increased	0 (0)	1 (11)	1 (5)

CCP = Cyclic Citrullinated Peptide; TNF = Tumor Necrosis Factor; PCOS = Polycystic Ovary Syndrome; N/A = Not Applicable

*1 patient with both Mi2 and P155/140, 1 patient with both Mi2 and MJ

### Pre-post differences

Twenty of the twenty-one participants (95.2%) completed both pre- and post-participation questionnaires, with pre-participation questionnaires being completed an average of 42.8 days prior to the first session (range 3-102) and post-participation questionnaires being completed an average of 25.3 days following the final session (range 1–77). 33% of participants had elevated GAD-7 scores, with five participants experiencing mild anxiety and two participants experiencing moderate anxiety. Wilcoxon signed-rank test of pre- and post-participation GAD-7 scores for those with abnormal pre-participation scores indicated a non-significant difference between before (median 7, *n* = 7) and after (median = 3, *n* = 7) (p-value 0.0568). 33% of participants had elevated PHQ-8 scores, with six participants experiencing mild depression and one participant experiencing moderate depression. Wilcoxon signed-rank test of pre- and post-participation PHQ-8 scores for those with abnormal pre-participation scores indicated a non-significant large difference between before (median 5.5, *n* = 6) and after (median = 4, *n* = 6) (p-value 0.171). Table 2 demonstrates disease activity measures documented prior to and following completion of the narrative medicine intervention, and Table 2 demonstrates pre- and post-participation questionnaire responses with median scores, ranges and Wilcoxon-signed rank test results. There were no statistically significant differences when comparing pre- and post-participation questionnaire responses between patients with JDM versus those with JIA. There were no statistically-significant difference between pre-participation and post-participation questionnaires for GAD-7, PHQ-8, PROMIS Depression, CSSQ, and CATIS scales. Total time between pre- and post-participation completion was 139 days on average (range 74–197).

**Table 2:** Pre-participation and post-participation disease measures of physician-reported global and myositis measures for patients with juvenile idiopathic arthritis and juvenile dermatomyositis

Measure	Pre-Participation N (%)			Post-Participation N (%)		
	JDM	JIA	All	JDM	JIA	All
Physician-Reported Global						
Not documented	2 (16)	1 (11)	3 (14)	4 (33)	3 (33)	7 (33)
0/10	6 (50)	3 (33)	9 (43)	5 (42)	4 (44)	9 (43)
1/10	3 (24)	1 (11)	4 (19)	2 (16)	1 (11)	3 (14)
2/10	1 (8)	0 (0)	1 (5)	1 (8)	0 (0)	1 (5)
3/10	0 (0)	3 (33)	3 (14)	0 (0)	1 (11)	1 (5)
4/10	0 (0)	1 (11)	1 (5)	0 (0)	0 (0)	0 (0)
MMT8						
Not documented	2 (16)			3 (24)		
144	1 (8)			0 (0)		
145	0 (0)			1 (8)		
147	1 (8)			1 (8)		
149	4 (33)			1 (8)		
150	4 (33)			6 (50)		
CMAS						
Not documented	4 (33)			4 (33)		
42	1 (8)			0 (0)		
44	0 (0)			1 (8)		
47	1 (8)			1 (8)		
48	0 (0)			1 (8)		
50	1 (8)			0 (0)		
51	0 (0)			1 (8)		
52	5 (42)			4 (33)		
CDASI Activity						
Not documented	2 (16)			4 (33)		
0/96	6 (50)			5 (42)		
1/96	4 (33)			1 (8)		
2/96	0 (0)			1 (8)		
3/96	0 (0)			1 (8)		
CDASI Damage						
Not documented	2 (16)			4 (33)		
0/32	9 (75)			5 (42)		
1/32	1 (8)			2 (16)		
3/32	0 (0)			1 (8)		

JDM = Juvenile Dermatomyositis. JIA = Juvenile Idiopathic Arthritis. MMT8 = Manual Muscle Testing-8. CMAS = Childhood Myositis Assessment Scale. CDASI = Cutaneous Dermatomyositis Disease Area and Severity Index

**Table 3:** Wilcoxon signed-rank test of pre-participation and post-participation questionnaires for patients with juvenile idiopathic arthritis and juvenile dermatomyositis

Measure	Pre-Participation median (range) (*n* = 21)			Post-Participation median (range) (*n* = 20)			*P*-Value		
	JDM	JIA	All	JDM	JIA	All	JDM	JIA	All
GAD7	2 (0–10)	3.5 (0–13)	3 (0–13)	1.5 (0–5)	2.5 (0–13)	2 (0–13)	0.219	0.203	0.070
PHQ-8	2.5 (0–7)	3.5 (0–13)	3 (0–13)	1 (0–10)	2 (0–9)	2 (0–10)	0.833	0.394	0.317
PROMIS Depression	45.9 (35.9–57.6)	48.1 (35.9–62.5)	45.9 (35.9, 62.5)	45.9 (35.9–57.8)	48.1 (35.9–64)	48.1 (35.9, 64)	0.695	0.575	0.948
CATIS	45.5 (36–58)	39.5 (24–55)	42 (24, 58)	45.5 (31–59)	42 (31–52)	43.5 (31, 59)	0.637	0.674	0.950
CSSQ	2.5 (1–16)	4 (0–8)	3 (0, 16)	3 (0–10)	6 (0–14)	4 (0, 14)	0.964	0.090	0.314

DM = Juvenile Dermatomyositis. JIA = Juvenile Idiopathic Arthritis. PHQ-8 = Patient Health Questionnaire-8. GAD-7=Generalized Anxiety Disorder-7. CATIS=Childhood Attitude Towards Illness Scale. CSSQ=CoVID Student Stress Questionnaire

## Discussion

Participant attendance and engagement with each of the six narrative medicine sessions suggests feasibility of implementing a narrative-based intervention for patients with JDM and JIA. While there were no statistically significant changes in mental health questionnaires, this is likely attributed to the small study population. Other factors that may have resulted in non-significant change in reported mental health outcomes include heterogeneous age-based population and incomplete participation in all of the narrative medicine sessions.

Compared to other studies in similar populations, rates of anxiety and depression shown in our study align with previous studies, demonstrating approximately one third of participants had elevated levels of anxiety and depression [3–6]. This comparison, however, should be interpreted with caution recognizing potential differences in methodology and patient populations. The absence of patients with severe anxiety and depression may suggest a selection bias, recognizing those with severe mental health distress may not have energy or capability to join and participate in narrative medicine sessions. Analysis of those with initially elevated levels of anxiety and depression suggest a signal towards improvement of this sub-population, suggesting future narrative medicine interventions should consider targeting those with already elevated levels of anxiety and depression. These studies should account for disease activity when interpreting any changes in anxiety or depression. While there are no clear minimally clinically important differences (MCIDs) established in the pediatric population, adult studies suggest average MCID estimates are 3.7 points for PHQ-9 and 3.3 points for GAD-7 [20]. While application of these numbers to pediatric populations should be interpreted with caution, a shift in the GAD-7 median from 7 to 3 for those with abnormal pre-participation scores does suggest clinical relevance.

Importantly, while interpretation of disease activity is limited in this small sample size, disease activity over the time course of the study showed patients with JIA had worse disease activity at the onset of the study requiring initiation of medications, whereas nine patients with JDM had no change in medications and two patients with JDM were able to wean down on their medications, as shown in Table 1. This discrepancy advocates for caution when comparing the JIA and JDM cohorts, recognizing disease activity can play a critical role in the mental health of participants. While the intervention period was limited to a 3-month interval so as to minimize potential external influence on mental health outcomes such as medication changes or life events, the above medication changes during the intervention period do suggest future studies should consider an even shorter intervention length.

To date, there is only one study investigating narrative medicine-based interventions within patients with pediatric rheumatologic conditions. That study implemented a single narrative medicine-based intervention and demonstrated feasibility of an intervention for pediatric rheumatologic diseases [10]. This prior study, however, included all-comers without dividing patients by disease process, all female participants including seven patients with lupus, two patients with juvenile dermatomyositis, one patient with systemic sclerosis, two patients with juvenile idiopathic arthritis, one patient with Polyarteritis Nodosa and one patient with amplified musculoskeletal pain syndrome. Additionally, the prior study had only one session involved, whereas the current study demonstrates continued adherence with a longitudinal intervention including 6 sessions. While there is limited data integrating narrative medicine for youth living with chronic conditions, a systematic review of the literature demonstrates story-based interventions offer improvement in distress and depressive symptoms for youth living with chronic health conditions [21–23]. Other studies investigating narrative medicine impact on patients living with chronic disease have showed decreased HbA1C for adults living with diabetes as well as mitigated posttraumatic stress disorder (PTSD) symptoms for those living with PTSD, demonstrating the power narrative medicine can have on participants [24,25].

While the current study was completed 3.5 years after the onset of the COVID-19 pandemic, elevated scores of COVID stress demonstrate ongoing distress related to COVID among patients with JIA and JDM. Despite the shift in societal expectations and masking requirements over recent years, those living with chronic diseases requiring immunosuppressive agents may continue to experience ongoing distress related to COVID. This ongoing and potentially under-recognized distress suggests a need for additional supportive resources for patients to cope with ongoing psychological distress.

Recognizing the significant barriers to access for care of mental health-related support, this narrative medicine approach offers a creative and novel adjunct intervention that can help address mental health-related concerns for patients with chronic disease. This potential impact is supported further by patient and facilitator feedback that emphasize the positive influence this intervention had for participants. While narrative medicine does not replace professional mental health support, it may include social support missing in more traditional outpatient mental health models, thus offering a supplemental therapeutic option for patients with mental health distress. Other important adjunctive potential therapeutic interventions include animal-assisted interventions, art, play, music and dance therapy, mindfulness, and yoga among others [6].

As with any study, it is important to acknowledge potential challenges and barriers to study implementation and scalability. While the current study uses video-based interface of Zoom to maximize ease of access and convenience for participation, there is importantly a potential limitation of future application for individuals who do not have access to video-based technology. In the current study, families were able to join the sessions if needed over the phone without video access to maximize ability to participate. Given the intervention was Zoom-based with a risk of inattention of participants, art supplies were mailed to patients to help mitigate this potential challenge. Even with these interventions, the study did not achieve 100% session adherence. Potential ongoing areas to address for future studies include offering multiple times for each session to accommodate life-related events, school activities, etc. that may coincide with the narrative-based session timing. Keeping feasibility in mind, cost should be accounted for in future larger-scale sessions, with consideration of implementing a similar study without mailed art supplies to allow for scalability. Age-validation for anxiety and depression questionnaires is an important consideration that future studies should take into account when adjusting larger-scale interventions for young children.

Given the rarity of the diseases addressed in this study, it was difficult to recruit large numbers of participants from a single site. This pilot study can serve as a basis for future multi-center collaborative research investigating the potential application of narrative medicine to support the mental health of our pediatric rheumatology patients. Additionally, while 95% survey completion was attained, delay in incentive payments may have resulted in decreased survey response. The financial compensation for participation and survey completion may also have introduced selection bias. Future studies looking to complete similar interventions on a larger scale should consider scalability of facilitator training, cost-effectiveness, and potential integration into routine clinical care.

There may be a potential selection bias for participants, as older patients were diagnosed more remotely from participation when compared to younger patients. One potential explanation is that newly diagnosed adolescents may be less comfortable talking with others about their diagnosis when compared with adolescents who have been coping with the illness for a longer period of time. Future studies should consider investigating potential barriers to participation for newly diagnosed adolescents and integrate them in the narrative medicine intervention if possible to mitigate bias. Additionally, future studies should document reasoning behind refusal of participation so as to provide important insights into the feasibility and acceptability of the intervention.

As with many mental health interventions, there was a risk that this study may have increased dysregulation or stress levels for participants. Participants had the ability to be connected to a psychologist through SCH, and importantly the questionnaires allowed for screening, identification and referral for treatment of at-risk individuals who may otherwise not have been identified. Recognizing the age of participants, future studies should keep in mind potentially relevant age-related adaptations of narrative-based interventions.

## Conclusions

It is feasible to implement a narrative medicine intervention for pediatric patients with JIA and JDM. Future studies should target larger populations to understand the impact of narrative medicine interventions on anxiety, depression and attitude towards illness.

## Supplementary Information

Below is the link to the electronic supplementary material.

Supplementary Material 1

## Data Availability

The datasets used and analyzed during the current study are available from the corresponding author on reasonable request.

## Abbreviations

JDM: Juvenile Dermatomyositis
JIA: Juvenile Idiopathic Arthritis
GAD-7: Generalized Anxiety Disorder-7
PHQ-8: Patient Health Questionnaire-8
SCH: Seattle Children’s Hospital
PROMIS: Patient-Reported Outcomes Measurement Information System
CATIS: Childhood Attitude Toward Illness Scale
CSSQ: COVID Student Stress Questionnaire
PTSD: Posttraumatic stress disorder
