# Supplementary material for: Narrative medicine intervention for mental wellbeing in Juvenile Myositis and juvenile idiopathic arthritis
Source: Pediatr Rheumatol Online J. 2026 May 31;24:50. doi: 10.1186/s12969-026-01232-5 (PMC13435490; doi:10.1186/s12969-026-01232-5)
Supplement: Supplementary file 1 [file 12969_2026_1232_MOESM1_ESM.docx]

**Narrative Medicine Curriculum Outline**

**Curriculum Overview:**

**Overarching Themes/Project Goals:**

- To support mental health for participants
- To encourage a sense of community by creating connections that help participants know they’re not alone
- To help participants notice the beauty in themselves

**Demographics of Patients:**

Patients will be ages 6-21 and diagnosed with either juvenile idiopathic arthritis (JIA) or juvenile dermatomyositis (JDM).

Curriculum is divided by developmental age:

- Younger: Ages 6 to 11 years old
- Older: Ages 12 to 21 years old

**Session Overview:**

- Week 1: Introductions; Who are you?
- Week 2: Attention and Close Listening; Friends
- Week 3: Art and Photography
- Week 4: You and Your Provider
- Week 5: Medicine and Lab Draws
- Week 6: Resilience; Reflections

**Materials List:** To be gathered and delivered to participants prior to sessions.

- **Week 1:**
  - Journal (1)
  - Pencil (2)
  - I Am Poem (1)
- **Week 2:**
  - Rory’s Story Cubes (3)
  - Friend Poem (1)
- **Week 3:**
  - National Geographic Magazine (1)
  - Coloring Book Pages (3)
  - Blank pages (3)
  - Crayons (1 pack)
- **Week 4:**
  - Two Doctor Poem (1)
- **Week 5:**
  - Chocolate M&Ms (1)
  - Swedish Fish (1)
  - Comic Book pages (2)
- **Week 6:**
  - Postcard (1)

**Short Narrative/Description:**

The following curriculum is primarily targeted towards children with JIA and JDM to reflect on their medical experiences over 6 sessions. Instructions to the facilitator include quotes for leading each session as well as italicized instructions for guidance.

**Session 1: Ages 6-11**

**Session Goal**: To get to know one another and set group expectations.

**Gathering (15 min)**:

1. **12:00-12:15PM Introductions**: Facilitator 1: “Welcome to our first of six sessions where we will get to know each other, share stories, write and create together! My name is _____ *[insert your name here]* and I will be one of the facilitators of our group during these sessions. We also have ____ *[insert co-facilitator name here]* as our second facilitator during these sessions. We will be with you throughout this journey and guide everyone along the way.

Today, our focus will be getting to know each other! If your camera is off and you are willing and able to turn the camera on, please do so in order for us to see each other. To start, please share your name, how old you are, what your diagnosis is and how many years you have been diagnosed if you know or remember. Before we pass on to the next person, please tell us something you love about yourself (do you have a big heart? Do you like to laugh?), and share one action that is representative of yourself. For example, if you have a big heart, you can hold your hands up to your heart [*gesture hands to heart for demonstration purposes*]. Teach your action (what we’ll call our secret handshake) to the others in the group so we can remember! After you share your name and secret handshake, we’ll all say together ‘Welcome ______ [*insert name of a participant]*’ and use the secret handshake as we welcome them to the group.”

*[Have each member of the group share their name, background and ‘secret handshake.’ When everyone has shared, move on to the next section.]*

**Group Formation (10 min):**

2. **12:15-12:25 Set Expectations:** Facilitator 2: “Now that we’ve all done introductions, it’s time to talk about rules for these sessions so we all know it’s a safe space to talk with each other. We want to make sure everyone feels respected in our group. We will write these rules down so we can look back at them each session. *[Facilitator 1 pulls up a blank Word document to be saved for future sessions. While participants speak, type up their thoughts/rules in the word document for future reference.]* Can anyone think of some rules that can help make sure we all feel safe and respected?”

*[If needed, prompt examples of expectations with the following:*

*-Do not speak when someone else is speaking.*

*-Don’t leave the room during these sessions unless your parent or family member tells you to do so or there is an emergency.*

*-What’s said in the zoom room stays in the zoom room. It’s okay to talk with your parents or caregivers about what we talk about here.*

*-It’s okay to have different ideas or feelings.*

*-If you feel comfortable, keep your camera on so we can see each other.*

*-Try to join the sessions on time to not miss anything!]*

3. **12:25-12:30 Art Packet Review:** Facilitator 1: “You all should have received a packet in the mail with some fun art supplies that we’ll be using during our sessions. Each session, we’ll tell you which supplies you’ll need so you should try to bring that package or have access to the supplies for each session. You’ll find a journal with pencils and crayons that you can use during and between sessions to write or draw what comes to mind. The envelopes are labeled by week, so keep those envelopes handy for when the week’s session comes! This week, you’ll need the envelope labeled ‘Week 1.’ If you haven’t gotten the supplies yet or don’t have them with you, that’s okay. Let us know or have your caregivers tell Dr. Lanis so we can get them to you for the next session.”

**Regulation Break (3 min):**

4. **12:30-12:33PM** **Stretch Break:** Facilitator 2: “You guys have made it to the halfway point, which means it’s time for our stretch break! At each session, we will take 3 minutes to stretch together and take a break from having to focus. Everyone can get out of their chair/seated position, stand up and when you’re ready, we’re going to REACH for the sky! Then REACH to the ground. Reach to the LEFT, then reach to the RIGHT. Stand straight up, nice and tall, and time for jumping jacks! Let’s do 10 jumping jacks all together. If you’re not feeling up for jumping jacks today, I invite you to march in place while we count to 10. *[Count from 1 to 10 as the group does jumping jacks]*. And take a breath. You guys have done so well, I want you to take a deep breath, in and out. And when you’re ready, sit back down in your chair/seated position, and we’ll jump right into our next part. *[If anyone needs to use the restroom, it is okay to pause here to allow them to use the restroom and wait to start the next prompt until they rejoin the group if possible.]*

**Narrative Exercise: Writing Prompt (20 minutes)**

5. **12:33-12:53 Narrative Prompt:** Facilitator 2: And now, we’ll start with our writing and drawing for the day! In your packet, you’ll find an envelope for week 1. In there, you’ll see a paper that starts with the words ‘I Am.’ We’re going to take 5 minutes to fill in the blanks and when everyone is done, we’ll share together. You can write words, full sentences, draw pictures, whatever feels most comfortable to you. This will hopefully give you a chance to share who you are with the group. During this time, I will read each sentence line-by-line, and allow you 15 seconds per line to write or draw a response. If you have difficulty reading the words, you can listen to my words to know what to write or draw in response. If you prefer to turn your camera off for the writing you may, but we ask that you turn the camera back on at the end of the writing period. *[Facilitator 2 will keep track of the 15-second increments while reading the following ‘I am’ prompts. Facilitator 1 is invited to try to write responses the poem, as sharing of facilitator writing may help prompt discussion through silence in the next phase.]*

*[Facilitator 2 reads the following, allowing 30 seconds between each phrase]* I am, I wonder, I hear, I see, I want, I am, I pretend, I feel, I touch. *[If time allows, you can continue with the following phrases]* I worry, I cry, I am, I understand, I say, I dream, I try, I hope, I am.

Take 15-20 seconds to finish up any drawings, words or sentences that you’d like to finish.

And with that, we have come to the end of our writing and drawing period. Try to finish the last sentence, draw that last line, and when you’re ready, look up (or turn on your cameras if they were off) so we can see you. As this was our first writing time together, I’d like to pause and check in. How was that? How did it go trying to fill in the words? *[Allow for silence if needed to encourage 1-2 speakers to share about the experience. If no one shares, facilitator can share their own experience of trying to fill in the poem.]*

Now that we’ve talked about how that went, let’s see if anyone is willing to share what they wrote or drew. As we share, I’d like to remind everyone that this is a safe space. You can share part or all of what you’d written or drawn. There is no judgment of what you’ve written, we’re here to support each other in this process. Who would like to go first? [*If no one is willing to share first, facilitator 1 can share their own writing. If there still is difficulty for participants to share their whole poem, facilitator 2 may go through the following line by line in the poem to ask what members of the group wrote for each one, for example “What did people write for the first I am?’ Did you write something different for the next 3 ‘I am’s?’]*

a. I am, I wonder, I hear, I see, I want, I am, I pretend, I feel, I touch, I worry, I cry, I am, I understand, I say, I dream, I try, I hope, I am

**Closing (7 min):**

6. **12:53-1PM Grounding Activity**: Facilitator 1: “Thank you all for sharing. At the end of every session, we will close with a short breathing activity. Hopefully this will help you get back to your day’s activities. First, we’ll watch this video to learn how to do the breathing activity together. Then, we’ll each take a few minutes and do the breathing all together.

*[Facilitator 2 pulls up below video and plays it for the group when Facilitator 1 is done speaking:]*

5 finger breathing <https://www.youtube.com/watch?v=HQVZgpyVQ78>

Now that you’ve seen the video, let’s all take a minute and do the exercise together. Take your finger to the base of your thumb, and trace the edge, breathing in, then breath out. Breath in, breath out. *[Continue verbalizing to breath in and out while moving fingers to replicate video.*] When you’re ready, shake your hand, then your arm, then your whole body if you’d like. And when you’ve shaken everything you needed to shake out, come back to stillness, together. Thank you for your time with us today, and we’ll see you in 2 weeks, same time same place. If you think you won’t be able to make it, have your parent or caregiver reach out to Dr. Lanis to discuss next steps. Thanks and have a great rest of the day!

**Session 1: Ages 12-21**

**Session Goal**: To get to know one another and set group expectations.

**Gathering (15 min)**:

1. **12:00-12:15PM Introductions**: Facilitator 1: “Welcome to our first of six sessions where we will get to know each other, share stories, write and create together! My name is _____ *[insert your name here]* and I will be one of the facilitators of our group during these sessions. We also have ____ *[insert co-facilitator name here]* as our second facilitator during these sessions. We will be with you throughout this journey and guide everyone along the way.

Today, our focus will be getting to know each other! If your camera is off and you are willing and able to turn the camera on, please do so in order for us to see each other. To start, please share your name, how old you are, what your diagnosis is and how many years you have been diagnosed. Before we pass on to the next person, please tell us something you love about yourself (do you have a big heart? Do you like to laugh?), and share one action that is representative of yourself. For example, if you have a big heart, you can hold your hands up to your heart [*gesture hands to heart for demonstration purposes*]. Teach your action (what we’ll call our secret handshake) to the others in the group so we can remember! After you share your name and secret handshake, we’ll all say together ‘Welcome ______ [*insert name of a participant]*’ and use the secret handshake as we welcome them to the group.”

*[Have each member of the group share their name, background and ‘secret handshake.’ When everyone has shared, move on to the next section.]*

**Group Formation (10 min):**

2. **12:15-12:25 Set Expectations:** Facilitator 2: “Now that we’ve all done introductions, it’s time to talk about rules for these sessions so we all know it’s a safe space to talk with each other. We want to make sure everyone feels respected in our group. We will write these rules down so we can look back at them each session. *[Facilitator 1 pulls up a blank Word document to be saved for future sessions. While participants speak, type up their thoughts/rules in the word document for future reference.]* Can anyone think of some rules that can help make sure we all feel safe and respected?”

*[If needed, prompt examples of expectations with the following:*

*-Do not speak when someone else is speaking.*

*-Don’t leave the room during these sessions unless your parent or family member tells you to do so or there is an emergency.*

*-What’s said in the zoom room stays in the zoom room. It’s okay to talk with your parents or caregivers about what we talk about here.*

*-If you feel comfortable, keep your camera on so we can see each other.*

*-It’s okay to have different ideas or feelings.*

*-Try to join the sessions on time to not miss anything!]*

3. **12:25-12:30 Art Packet Review:** Facilitator 1: “You all should have received a packet in the mail with some fun art supplies that we’ll be using during our sessions. Each session, we’ll tell you which supplies you’ll need so you should try to bring that package or have access to the supplies for each session. You’ll find a journal with pencils and crayons that you can use during and between sessions to write or draw what comes to mind. The envelopes are labeled by week, so keep those envelopes handy for when the week’s session comes! This week, you’ll need the envelope labeled ‘Week 1.’ If you haven’t gotten the supplies yet or don’t have them with you, that’s okay. Let us know or have your caregivers tell Dr. Lanis so we can get them to you for the next session.”

**Narrative Exercise: Writing Prompt (20 minutes)**

4. **12:30-12:50 Narrative Prompt:** Facilitator 2: And now, we’ll start with our writing prompts for the day! In your packet, you’ll find an envelope for week 1. In there, you’ll see a paper that starts with the words ‘I Am.’ We’re going to take 5 minutes to fill in the blanks and when everyone is done, we’ll share together. You can write words, full sentences, draw pictures, whatever feels most comfortable to you. We will let you know when you have 1 minute left in the writing period. This will hopefully give you a chance to share who you are with the group. When you’re ready, you may begin. *[Allow 5 minutes of writing, Facilitator 1 keeps time and notifies the group when there is 1 minute remaining in the writing period. Both facilitator 1 and 2 are invited to try to write the poem for themselves as well, as sharing of their own writing may help facilitate discussion through silence in the next phase.]*

*[When there is 1 minute left of writing, Facilitator 1 state the following:]* You have 1 minute left for writing.

*[After 5 minutes of writing, Facilitator 1 states the following:]* And with that, we have come to the end of our writing period. Try to finish the last sentence, draw that last line, and when you’re ready, look up (or turn on your cameras if they were off) so we can see you.

*Facilitator 2:* As this was our first writing time together, I’d like to pause and check in. How was that? How did it go trying to fill in the words? *[Allow for silence if needed to encourage 1-2 speakers to share about the experience. If no one shares, facilitators can share their own experience of trying to fill in the poem.]*

Now that we’ve talked about how that went, let’s see if anyone is willing to share what they wrote. As we share, I’d like to remind everyone that this is a safe space. You can share part or all of what you’d written or drawn. There is no judgment of what you’ve written, we’re here to support each other in this process. Who would like to go first? [*If no one is willing to share first, facilitator 1 can share their writing. If there still is difficulty for participants to share their whole poem, facilitator 2 may go through the following line by line in the poem to ask what members of the group wrote for each one, for example “What did people write for the first I am?’ Did you write something different for the next 3 ‘I am’s?’]*

a. I am, I wonder, I hear, I see, I want, I am, I pretend, I feel, I touch, I worry, I cry, I am, I understand, I say, I dream, I try, I hope, I am

**Closing (10 min):**

5. **12:50-1PM Grounding Activity**: Facilitator 1: Thank you all for sharing. Without needing to share with the group, we’re going to write a few questions in the chat for you to think about as you reflect on today’s experience. These questions are also listed on one of the cards you should all have in your week 1 envelope that we’ll refer back to at the end of each session. You can reflect on these questions while we do our breathing activity to end the session, or if you prefer you can write them down in your journal after we finish today’s session. We hope these questions will prompt reflections on today’s session. The questions we offer are as follows:

*[Facilitator 2 writes the following in the chat box while Facilitator 1 is speaking. Facilitator 1 pauses for a few seconds between questions to allow for time for the participants to process.]*

I wonder, what did you notice?

I wonder, what was the most important part of today’s group for you?

I wonder, what will you remember tomorrow?

I wonder, what do you wonder about next session?

At the end of every session, we will close with a short breathing activity. You are welcome to ponder the questions we just discussed as we go through this breathing activity, and think to yourself what your answers are. Hopefully this breathing activity will help you get back to your day’s activities. First, we’ll watch this video to learn how to do the breathing activity together. Then, we’ll each take a few minutes and do the breathing all together.

*[Facilitator 2 pulls up below video while Facilitator 1 is speaking:]*

5 finger breathing <https://www.youtube.com/watch?v=HQVZgpyVQ78>

Now that you’ve seen the video, let’s all take a minute and do the exercise together. Take your finger to the base of your thumb, and trace the edge, breathing in, then breath out. Breath in, breath out. *[Continue verbalizing to breath in and out while moving fingers to replicate video.]* When you’re ready, shake your hand, then your arm, then your whole body if you’d like. And when you’ve shaken everything you needed to shake out, come back to stillness, together.

Thank you for your time with us today, and we’ll see you in 2 weeks, same time same place. If you think you won’t be able to make it, have your parent or caregiver reach out to Dr. Lanis to discuss next steps. Thanks and have a great rest of the day!

**Session 2: Ages 6-11**

**Session Goal**: To encourage attention and close listening as well as group cohesion, and to help participants learn to talk about disease narratives with friends.

**Gathering and Expectations (15 min)**:

1. **12:00-12:10PM Gathering**: Facilitator 1: “Welcome to our second of six sessions where we will get to know each other, share stories, write and create together! For those who may not remember or may have missed our first session, my name is _____ *[insert your name here]* and I will be one of the facilitators of our group during these sessions. We also have ____ *[insert co-facilitator name here]* as our second facilitator during these sessions. We will be with you throughout this journey and guide everyone along the way.

Today, our focus will be how to share stories and listen to our friends in the group. Just like last time, we’ll start with re-introductions so folks can try to remember names and secret handshakes! If your camera is off and you are willing and able to turn the camera on, please do so in order for us to see each other. For those who have been with us last time, can you please re-introduce yourself and share your name, something you love about yourself and your secret handshake?

For those who are new to the group, can you please share your name, how old you are, what your diagnosis is and how many years you have been diagnosed. Before we pass on to the next person, please tell us something you love about yourself (do you have a big heart? Do you like to laugh?), and share one action that is representative of yourself. For example, if you have a big heart, you can hold your hands up to your heart [*gesture hands to heart for demonstration purposes*]. Teach your action (what we’ll call our secret handshake) to the others in the group so we can remember! After you share your name and secret handshake, we’ll all say together ‘Welcome ______ [*insert name of a participant]*’ and use the secret handshake as we welcome them to the group.”

*[Have each member of the group share their name and ‘secret handshake.’ When everyone has shared, move on to the next section.]*

2. **12:10-12:15 Set Expectations:** Facilitator 2: “Now that we’ve all done introductions, it’s time to remind each other of our rules for these sessions so we all know it’s a safe space to talk with each other. We want to make sure everyone feels respected in our group. *[Facilitator 1 pulls up Word document from prior session with rules. Facilitator 2 reads prior rules created by the group.]* Now that we’ve read through the rules together, we can get started with our first activity!”

*[If needed or no Word document available, can review expectations with the following:*

*-Do not speak when someone else is speaking.*

*-Don’t leave the room during these sessions unless your parent or family member tells you to do so or there is an emergency.*

*-What’s said in the zoom room stays in the zoom room. It’s okay to talk with your parents or caregivers about what we talk about here.*

*-It’s okay to have different ideas or feelings.*

*-If you feel comfortable, keep your camera on so we can see each other.*

*-Try to join the sessions on time to not miss anything!]*

**Group Formation (20 min):**

3. **12:15-12:35 Attention and Close Listening:** Facilitator 1: Now that we’ve reminded ourselves of the rules for today, let’s get started with our activity! You’ll find in your packet for week 2 some dice, which are Rory’s Story Cubes. Each person should have 3 dice. You’ll see that these are very special dice, as each die has photos instead of numbers on every face. For this activity, we’ll take turns. When it’s your turn, you’ll roll the dice. Your job is to start a story with “Once upon a time,” and create a sentence that brings in one of the photos your dice rolled. If you have a photo you don’t understand or know what it is, you can re-roll that die. Once you’ve finished your sentence, the next person will roll their dice and CONTINUE the first story, again using one of the 3 photos within the story. We’ll start with 1 round for everyone to get a turn and see how much time we have to do a second round. We’ll have ____ *[Facilitator 2’s name]* start to give an example for everyone. Once you’ve shared your line in the story, you can call on the next person to roll their dice and share their line in the story. It’s very important to listen and pay attention to what everyone is saying so you can know where we are in the story. *[Facilitator 2 can start the round by rolling the dice from their packet. Go around the room, if participants are unsure of how to proceed offer encouragement and call on participants if they don’t call on each other to share a line in the story. If participants do not have dice available, facilitator can roll their own dice and offer options to participant for them to choose a die to prompt their story line.]*

**Regulation Break (3 min):**

4. **12:35-12:38PM** **Stretch Break:** Facilitator 2: “You guys have made it to the halfway point, which means it’s time for our stretch break! Everyone can get out of their chair/seated position, stand up and when you’re ready, we’re going to REACH for the sky! Then REACH to the ground. Reach to the LEFT, then reach to the RIGHT. Stand straight up, nice and tall, and time for jumping jacks! Let’s do 10 jumping jacks all together. If you’re not feeling up for jumping jacks today, I invite you to march in place while we count to 10. *[Count from 1 to 10 as the group does jumping jacks]*. And take a breath. You guys have done so well, I want you to take a deep breath, in and out. And when you’re ready, sit back down in your chair/seated position, and we’ll jump right into our next part. *[If anyone needs to use the restroom, it is okay to pause here to allow them to use the restroom and wait to start the next prompt until they rejoin the group if possible.]*

**Narrative Exercise: Writing Prompt (15 minutes)**

5. **12:38-12:53PM Poem:** Facilitator 2: **“**Welcome back! We’re now going to shift gears a bit and read a poem together. You’ll find this poem in the envelope for Week 2 entitled “Friend Poem” if you’d like to read along. I’ll read it out loud to the group. As you listen, I want you to think, what do you think the author felt writing this poem?

*[Facilitator 1 pulls up powerpoint with poem for people to see.]*

Now that we’ve read the poem together, I want you to take your journal and pencil/crayons. With your pencil/crayons, write to or draw for any of your friends or a family member who haven’t been feeling well recently. What would you write or draw to help them feel better? You’ll have 5 minutes to write or draw. When you’re done writing/drawing, we’ll share what we wrote or drew together. We’ll let you know when you have 1 minute left. You may begin!”

*[Facilitator 1 keeps track of time. When there is 1 minute remaining, facilitator 1 states the following:]* You have 1 minute remaining.

*[At the end of the 5 minutes, Facilitator 1 notifies the group that writing time is completed. Facilitator 2 then continues:]* Now that time is up, it’s time to share! Who would like to share what they wrote or drew? Did you draw or write what you would want a friend to draw or say for you if you were sick? If not, what would you want someone to write or draw for you?

**Closing (7 min):**

6. **12:53-1PM Grounding Activity**: Facilitator 1: “Thank you all for sharing. At the end of every session, we will close with a short breathing activity. Hopefully this will help you get back to your day’s activities. First, we’ll watch this video to learn how to do the breathing activity together. Then, we’ll each take a few minutes and do the breathing all together.

*[Facilitator 2 pulls up below video and plays it for the group when Facilitator 1 is done speaking:]*

5 finger breathing <https://www.youtube.com/watch?v=HQVZgpyVQ78>

Now that you’ve seen the video, let’s all take a minute and do the exercise together. Take your finger to the base of your thumb, and trace the edge, breathing in, then breath out. Breath in, breath out. *[Continue verbalizing to breath in and out while moving fingers to replicate video.*] When you’re ready, shake your hand, then your arm, then your whole body if you’d like. And when you’ve shaken everything you needed to shake out, come back to stillness, together. Thank you for your time with us today, and we’ll see you in 2 weeks, same time same place. If you think you won’t be able to make it, have your parent or caregiver reach out to Dr. Lanis to discuss next steps. Thanks and have a great rest of the day!

**Session 2: Ages 12-21**

**Session Goal**: To encourage attention and close listening as well as group cohesion, and to help participants learn to talk about disease narratives with friends.

**Gathering and Expectations (15 min)**:

1. **12:00-12:10PM Gathering**: Facilitator 1: “Welcome to our second of six sessions where we will get to know each other, share stories, write and create together! For those who may not remember or may have missed our first session, my name is _____ *[insert your name here]* and I will be one of the facilitators of our group during these sessions. We also have ____ *[insert co-facilitator name here]* as our second facilitator during these sessions. We will be with you throughout this journey and guide everyone along the way.

Today, our focus will be how to share stories and listen to our friends in the group. Just like last time, we’ll start with re-introductions so folks can try to remember names and secret handshakes! If your camera is off and you are willing and able to turn the camera on, please do so in order for us to see each other. For those who have been with us last time, can you please re-introduce yourself and share your name, something you love about yourself and your secret handshake?

For those who are new to the group, can you please share your name, how old you are, what your diagnosis is and how many years you have been diagnosed. Before we pass on to the next person, please tell us something you love about yourself (do you have a big heart? Do you like to laugh?), and share one action that is representative of yourself. For example, if you have a big heart, you can hold your hands up to your heart [*gesture hands to heart for demonstration purposes*]. Teach your action (what we’ll call our secret handshake) to the others in the group so we can remember! After you share your name and secret handshake, we’ll all say together ‘Welcome ______ [*insert name of a participant]*’ and use the secret handshake as we welcome them to the group.”

*[Have each member of the group share their name and ‘secret handshake.’ When everyone has shared, move on to the next section.]*

2. **12:10-12:15 Set Expectations:** Facilitator 2: “Now that we’ve all done introductions, it’s time to remind each other of our rules for these sessions so we all know it’s a safe space to talk with each other. We want to make sure everyone feels respected in our group. *[Facilitator 1 pulls up Word document from prior session with rules. Facilitator 2 reads prior rules created by the group.]* Now that we’ve read through the rules together, we can get started with our first activity!”

*[If needed or no Word document available, can review expectations with the following:*

*-Do not speak when someone else is speaking.*

*-Don’t leave the room during these sessions unless your parent or family member tells you to do so or there is an emergency.*

*-What’s said in the zoom room stays in the zoom room. It’s okay to talk with your parents or caregivers about what we talk about here.*

*-It’s okay to have different ideas or feelings.*

*-If you feel comfortable, keep your camera on so we can see each other.*

*-Try to join the sessions on time to not miss anything!]*

**Group Formation (20 min):**

3. **12:15-12:35 Attention and Close Listening:** Facilitator 1: Now that we’ve reminded ourselves of the rules for today, let’s get started with our activity! You’ll find in your packet for week 2 some dice, which are Rory’s Story Cubes. Each person should have 3 dice. You’ll see that these are very special dice, as each die has photos instead of numbers on every face. For this activity, we’ll take turns. When it’s your turn, you’ll roll the dice. Your job is to start a story with “Once upon a time,” and create a sentence that brings in one of the photos your dice rolled. If you have a photo you don’t understand or know what it is, you can re-roll that die. Once you’ve finished your sentence, the next person will roll their dice and CONTINUE the first story, again using one of the 3 photos within the story. It’s very important to listen and pay attention to what everyone is saying so you can know where we are in the story. We’ll start with 1 round for everyone to get a turn and see how much time we have to do a second round. Who would like to start?

*[If participants are shy or unsure how to start, Facilitator 2 can start the round. If participants do not have dice available, facilitator can roll their own dice and offer options to participant for them to choose a die to prompt their story line.]*

**Narrative Exercise: Writing Prompt (18 minutes)**

4. **12:35-12:53PM Poem:** Facilitator 2: **“**Good job everyone! We’re now going to shift gears a bit and read a poem together. You’ll find this poem in the envelope for Week 2 entitled “Friend Poem” if you’d like to read along. I’ll read it out loud to the group. As you listen, I want you to think, what do you think the author felt writing this poem?

*[Facilitator 1 pulls up powerpoint with poem for people to see.]*

Now that we’ve read the poem together, I want you to take your journal and pencil/crayons. With your pencil/crayons, write to or draw for any of your friends or a family member who haven’t been feeling well recently. What would you write or draw to help them feel better? You’ll have 5 minutes to write or draw. When you’re done writing/drawing, we’ll share what we wrote or drew together. We’ll let you know when you have 1 minute left. You may begin!”

*[Facilitator 1 keeps track of time. When there is 1 minute remaining, facilitator 1 states the following:]* You have 1 minute remaining.

*[At the end of the 5 minutes, Facilitator 1 notifies the group that writing time is completed. Facilitator 2 then continues:]* Now that time is up, it’s time to share! Who would like to share what they wrote or drew? Did you draw or write what you would want a friend to draw or say for you if you were sick? If not, what would you want someone to write or draw for you?

**Closing (10 min):**

5. **12:50-1PM Grounding Activity**: Facilitator 1: Thank you all for sharing. Without needing to share with the group, we’re going to write a few questions in the chat for you to think about as you reflect on today’s experience. These questions are also listed on one of the cards you should all have in your week 2 envelope that we’ll refer back to at the end of each session. You can reflect on these questions while we do our breathing activity to end the session, or if you prefer you can write them down in your journal after we finish today’s session. We hope these questions will prompt reflections on today’s session. The questions we offer are as follows:

*[Facilitator 2 writes the following in the chat box while Facilitator 1 is speaking. Facilitator 1 pauses for a few seconds between questions to allow for time for the participants to process.]*

I wonder, what did you notice?

I wonder, what was the most important part of today’s group for you?

I wonder, what will you remember tomorrow?

I wonder, what do you wonder about next session?

At the end of every session, we will close with a short breathing activity. You are welcome to ponder the questions we just discussed as we go through this breathing activity, and think to yourself what your answers are. Hopefully this breathing activity will help you get back to your day’s activities. First, we’ll watch this video to learn how to do the breathing activity together. Then, we’ll each take a few minutes and do the breathing all together.

*[Facilitator 2 pulls up below video while Facilitator 1 is speaking:]*

5 finger breathing <https://www.youtube.com/watch?v=HQVZgpyVQ78>

Now that you’ve seen the video, let’s all take a minute and do the exercise together. Take your finger to the base of your thumb, and trace the edge, breathing in, then breath out. Breath in, breath out. *[Continue verbalizing to breath in and out while moving fingers to replicate video.]* When you’re ready, shake your hand, then your arm, then your whole body if you’d like. And when you’ve shaken everything you needed to shake out, come back to stillness, together.

Thank you for your time with us today, and we’ll see you in 2 weeks, same time same place. If you think you won’t be able to make it, have your parent or caregiver reach out to Dr. Lanis to discuss next steps. Thanks and have a great rest of the day!

**Session 3: Ages 6-11**

**Session Goal**: To engage with art and photography as a means of self-reflection.

**Gathering and Set Expectations (8 min)**:

1. **12:00-12:05PM Gathering**: Facilitator 1: “Welcome to our third of six sessions where we will get to know each other, share stories, write and create together! For those who may not remember or may have missed our first few sessions, my name is _____ *[insert your name here]* and I will be one of the facilitators of our group during these sessions. We also have ____ *[insert co-facilitator name here]* as our second facilitator during these sessions. We will be with you throughout this journey and guide everyone along the way.

Today, our focus will be how to use art and photography to share our experiences. Just like last time, we’ll start with re-introductions so folks can try to remember names and secret handshakes! If your camera is off and you are willing and able to turn the camera on, please do so in order for us to see each other. For those who have been with us last time, can you please re-introduce yourself and share your name, something you love about yourself and your secret handshake?

***[SKIP if everyone is already acquainted; add remaining time to second narrative prompt.* *Only proceed with the following if there are new members of the group that have not previously attended.]***

For those who are new to the group, can you please share your name, how old you are, what your diagnosis is and how many years you have been diagnosed. Before we pass on to the next person, please tell us something you love about yourself (do you have a big heart? Do you like to laugh?), and share one action that is representative of yourself. For example, if you have a big heart, you can hold your hands up to your heart [*gesture hands to heart for demonstration purposes*]. Teach your action (what we’ll call our secret handshake) to the others in the group so we can remember! After you share your name and secret handshake, we’ll all say together ‘Welcome ______ [*insert name of a participant]*’ and use the secret handshake as we welcome them to the group.”

*[Have each member of the group share their name and ‘secret handshake.’ When everyone has shared, move on to the next section.]*

2. **12:05-12:08 Set Expectations:** Facilitator 2: “Now that we’ve all done introductions, it’s time to remind each other of our rules for these sessions so we all know it’s a safe space to talk with each other. We want to make sure everyone feels respected in our group. *[Facilitator 1 pulls up Word document from prior session with rules. Facilitator 2 reads prior rules created by the group.]* Now that we’ve read through the rules together, we can get started with our first activity!”

*[If needed or no Word document available, can review expectations with the following:*

*-Do not speak when someone else is speaking.*

*-Don’t leave the room during these sessions unless your parent or family member tells you to do so or there is an emergency.*

*-What’s said in the zoom room stays in the zoom room. It’s okay to talk with your parents or caregivers about what we talk about here.*

*-It’s okay to have different ideas or feelings.*

*-If you feel comfortable, keep your camera on so we can see each other.*

*-Try to join the sessions on time to not miss anything!]*

**Narrative Exercise (22 min):**

3. **12:08-12:30 Narrative Art and Photography**: Facilitator 1: “For our first exercise today, we’re going to get our creative juices flowing. You’ll find in your packets a national geographic magazine, as well as some crayons. You’ll also find in blank paper and coloring book pages in the envelope labeled ‘Week 3.’ Today, we’re going to ask you to create a piece of art that represents yourself. What animal do you feel like when you’re angry, or happy, or sad? What color are you when you talk to your parents? What color are you when you talk to your friends? You can rip out a page from the national geographic magazine and use that as a basis, or color the coloring book pages with the colors you feel represent you. You can start from scratch with a blank page, and draw yourself as you see yourself. We’ll take 10 minutes to do the exercise, and then we’ll come back together to share what we’ve created. We’ll let you know when you have 1 minute left.”

*[Facilitator 2 keeps track of time, lets participants know when there is 1 minute remaining and when time is up.]*

*[Upon completion of the 10 minutes, Facilitator 1 continues:]* Now that you’ve finished your masterpieces, who would like to share first?

**Regulation Break (3 min):**

4. **12:30-12:33PM** **Stretch Break:** Facilitator 2: “You guys have made it to the halfway point, which means it’s time for our stretch break! Everyone can get out of their chair/seated position, stand up and when you’re ready, we’re going to REACH for the sky! Then REACH to the ground. Reach to the LEFT, then reach to the RIGHT. Stand straight up, nice and tall, and time for jumping jacks! Let’s do 10 jumping jacks all together. If you’re not feeling up for jumping jacks today, I invite you to march in place while we count to 10. *[Count from 1 to 10 as the group does jumping jacks]*. And take a breath. You guys have done so well, I want you to take a deep breath, in and out. And when you’re ready, sit back down in your chair/seated position, and we’ll jump right into our next part. *[If anyone needs to use the restroom, it is okay to pause here to allow them to use the restroom and wait to start the next prompt until they rejoin the group if possible.]*

**Narrative Exercise (20 min):**

5. **12:33-12:53: Photography:** Facilitator 2: “As we come back together, let’s get ready for our next activity for today. Before we get started, I’d like to introduce you to Angela Lundberg, a patient with rheumatoid arthritis who uses photography to process her experience with rheumatoid arthritis. *[Facilitator 1 shows slides from Week 3 in PowerPoint as below and plays the video, then moves to second slide as discuss photo prompts.]* Here are some of the photos she has taken of her experiences with RA. For this part of our session today, we invite you to choose a photo that speaks to you, and take 5 minute to write or draw in your journal. As you write or draw, think about how the photo makes you feel using your five senses. What do you see? What do you smell? What do you taste? What do hear? What words come to mind? You can draw a happy face, write a paragraph, a poem, a story, it can make sense or not, you don’t have to worry about it. Just keep your hand or pencil or crayon moving; if you’re writing and you get stuck keep writing the last word over and over until the next idea or thought comes to you. We’ll let you know when you have 1 minute left, and at the end of the 5 minutes, we will have 15 minutes and ask everyone to share their writings or drawings.”

*[Facilitator 1 brings up PowerPoint with these photos to share on the screen while Facilitator 2 is talking. Facilitator 1 keeps time and notifies the group when there is 1 minute left and when time is up. Both Facilitator 1 and 2 are invited to write responses that they can share with the group if there is silence or the group members are hesitant to share their writings.]*

*Photos courtesy of Angela Lundberg.*

“Our writing time is up together, which means it’s time to have space to share. It can be very scary to share your work and that’s okay, we’re here to support each other through the process. Tell us which image you chose, and you can choose a couple of words, a phrase or to share your whole writing with the group. If you prefer to pass, that’s okay, just say pass. We can go around again at the end if you feel comfortable to share as well.”

**Closing (7 min):**

6. **12:53-1PM Grounding Activity**: Facilitator 1: “Thank you all for sharing. At the end of every session, we will close with a short breathing activity. Hopefully this will help you get back to your day’s activities. First, we’ll watch this video to learn how to do the breathing activity together. Then, we’ll each take a few minutes and do the breathing all together.

*[Facilitator 2 pulls up below video and plays it for the group when Facilitator 1 is done speaking:]*

5 finger breathing <https://www.youtube.com/watch?v=HQVZgpyVQ78>

Now that you’ve seen the video, let’s all take a minute and do the exercise together. Take your finger to the base of your thumb, and trace the edge, breathing in, then breath out. Breath in, breath out. *[Continue verbalizing to breath in and out while moving fingers to replicate video.*] When you’re ready, shake your hand, then your arm, then your whole body if you’d like. And when you’ve shaken everything you needed to shake out, come back to stillness, together. Thank you for your time with us today, and we’ll see you in 2 weeks, same time same place. If you think you won’t be able to make it, have your parent or caregiver reach out to Dr. Lanis to discuss next steps. Thanks and have a great rest of the day!

**Session 3: Ages 12-21**

**Session Goal**: To engage with art and photography as a means of self-reflection.

**Gathering and Set Expectations (8 min)**:

1. **12:00-12:05PM Gathering**: Facilitator 1: “Welcome to our third of six sessions where we will get to know each other, share stories, write and create together! For those who may not remember or may have missed our first few sessions, my name is _____ *[insert your name here]* and I will be one of the facilitators of our group during these sessions. We also have ____ *[insert co-facilitator name here]* as our second facilitator during these sessions. We will be with you throughout this journey and guide everyone along the way.

Today, our focus will be how to use art and photography to share our experiences. Just like last time, we’ll start with re-introductions so folks can try to remember names and secret handshakes! If your camera is off and you are willing and able to turn the camera on, please do so in order for us to see each other. For those who have been with us last time, can you please re-introduce yourself and share your name, something you love about yourself and your secret handshake?

***[SKIP if everyone is already acquainted; add remaining time to second narrative prompt.* *Only proceed with the following if there are new members of the group that have not previously attended.]***

For those who are new to the group, can you please share your name, how old you are, what your diagnosis is and how many years you have been diagnosed. Before we pass on to the next person, please tell us something you love about yourself (do you have a big heart? Do you like to laugh?), and share one action that is representative of yourself. For example, if you have a big heart, you can hold your hands up to your heart [*gesture hands to heart for demonstration purposes*]. Teach your action (what we’ll call our secret handshake) to the others in the group so we can remember! After you share your name and secret handshake, we’ll all say together ‘Welcome ______ [*insert name of a participant]*’ and use the secret handshake as we welcome them to the group.”

*[Have each member of the group share their name and ‘secret handshake.’ When everyone has shared, move on to the next section.]*

2. **12:05-12:08 Set Expectations:** Facilitator 2: “Now that we’ve all done introductions, it’s time to remind each other of our rules for these sessions so we all know it’s a safe space to talk with each other. We want to make sure everyone feels respected in our group. *[Facilitator 1 pulls up Word document from prior session with rules. Facilitator 2 reads prior rules created by the group.]* Now that we’ve read through the rules together, we can get started with our first activity!”

*[If needed or no Word document available, can review expectations with the following:*

*-Do not speak when someone else is speaking.*

*-Don’t leave the room during these sessions unless your parent or family member tells you to do so or there is an emergency.*

*-What’s said in the zoom room stays in the zoom room. It’s okay to talk with your parents or caregivers about what we talk about here.*

*-It’s okay to have different ideas or feelings.*

*-If you feel comfortable, keep your camera on so we can see each other.*

*-Try to join the sessions on time to not miss anything!]*

**Narrative Exercise (22 min):**

3. **12:08-12:30 Narrative Art and Photography**: Facilitator 1: “For our first exercise today, we’re going to get our creative juices flowing. You’ll find in your packets a national geographic magazine, as well as some crayons. You’ll also find in blank paper and coloring book pages in the envelope labeled ‘Week 3.’ Today, we’re going to ask you to create a piece of art that represents yourself. What animal do you feel like when you’re angry, or happy, or sad? What color are you or what face do you wear when you talk to your parents? What color are you or what face do you wear when you talk to your friends, or another patient? You can rip out a page from the national geographic magazine and use that as a basis, or color the coloring book pages with the colors you feel represent you. You can start from scratch with a blank page, and draw yourself as you see yourself. We’ll take 10 minutes to do the exercise, and then we’ll come back together to share what we’ve created. We’ll let you know when you have 1 minute left.”

*[Facilitator 2 keeps track of time, lets participants know when there is 1 minute remaining and when time is up.]*

*[Upon completion of the 10 minutes, Facilitator 1 continues:]* Now that you’ve finished your masterpieces, who would like to share first?

**Narrative Exercise (20 min):**

4. **12:30-12:50: Photography:** Facilitator 2: “Great job everyone, thanks for sharing. Now that we’ve done some drawing, we’d like to spend some time writing today. But first, I’d like to introduce you to Angela Lundberg, a patient with rheumatoid arthritis who uses photography to process her experience with rheumatoid arthritis. *[Facilitator 1 shows slides from Week 3 in PowerPoint as below and plays the video, then moves to second slide as discuss photo prompts.]* Here are some of the photos she has taken of her experiences with RA. For this part of our session today, we invite you to choose a photo that speaks to you, and take 5 minute to write in your journal. As you write, think about how the photo makes you feel using your five senses. What do you see? What do you smell? What do you taste? What do hear? What words come to mind? You can write a paragraph, a poem, a story, it can make sense or not, you don’t have to worry about it. Just keep your hand or pencil moving; if you get stuck keep writing the last word over and over until the next idea or thought comes to you. We’ll let you know when you have 1 minute left, and at the end of the 5 minutes, we will have 15 minutes and ask everyone to share their writings.”

*[Facilitator 1 brings up PowerPoint with these photos to share on the screen while Facilitator 2 is talking. Facilitator 1 keeps time and notifies the group when there is 1 minute left and when time is up. Both Facilitator 1 and 2 are invited to write responses that they can share with the group if there is silence or the group members are hesitant to share their writings.]*

*Photos courtesy of Angela Lundberg.*

“Our writing time is up together, which means it’s time to have space to share. It can be very scary to share your work and that’s okay, we’re here to support each other through the process. Tell us which image you chose, and you can choose a couple of words, a phrase or to share your whole writing with the group. If you prefer to pass, that’s okay, just say pass. We can go around again at the end if you feel comfortable to share as well.”

**Closing (10 min):**

5. **12:50-1PM Grounding Activity**: Facilitator 1: Thank you all for sharing. Without needing to share with the group, we’re going to write a few questions in the chat for you to think about as you reflect on today’s experience. These questions are also listed on one of the cards you should all have in your packet that we’ll refer back to at the end of each session. You can reflect on these questions while we do our breathing activity to end the session, or if you prefer you can write them down in your notebook after we finish today’s session. We hope these questions will prompt reflections on today’s session. The questions we offer are as follows:

*[Facilitator 2 writes the following in the chat box while Facilitator 1 is speaking. Facilitator 1 pauses for a few seconds between questions to allow for time for the participants to process.]*

I wonder, what did you notice?

I wonder, what was the most important part of today’s group for you?

I wonder, what will you remember tomorrow?

I wonder, what do you wonder about next session?

At the end of every session, we will close with a short breathing activity. You are welcome to ponder the questions we just discussed as we go through this breathing activity, and think to yourself what your answers are. Hopefully this breathing activity will help you get back to your day’s activities. First, we’ll watch this video to learn how to do the breathing activity together. Then, we’ll each take a few minutes and do the breathing all together.

*[Facilitator 2 pulls up below video while Facilitator 1 is speaking:]*

5 finger breathing <https://www.youtube.com/watch?v=HQVZgpyVQ78>

Now that you’ve seen the video, let’s all take a minute and do the exercise together. Take your finger to the base of your thumb, and trace the edge, breathing in, then breath out. Breath in, breath out. *[Continue verbalizing to breath in and out while moving fingers to replicate video.]* When you’re ready, shake your hand, then your arm, then your whole body if you’d like. And when you’ve shaken everything you needed to shake out, come back to stillness, together.

Thank you for your time with us today, and we’ll see you in 2 weeks, same time same place. If you think you won’t be able to make it, have your parent or caregiver reach out to Dr. Lanis to discuss next steps. Thanks and have a great rest of the day!

**Session 4: Ages 6-11**

**Session Goal**: To reflect on the interaction between patients and their providers.

**Gathering and Set Expectations (5 min)**:

1. **12:00-12:05PM Gathering**: Facilitator 1: “Welcome to our fourth of six sessions where we will get to know each other, share stories, write and create together! As a reminder, my name is _____ *[insert your name here]* and I will be one of the facilitators of our group during these sessions. We also have ____ *[insert co-facilitator name here]* as our second facilitator during these sessions. We will be with you throughout this journey and guide everyone along the way.
2. **12:05-12:08 Set Expectations:** Facilitator 2: “Now that we’ve all done introductions, it’s time to remind each other of our rules for these sessions so we all know it’s a safe space to talk with each other. We want to make sure everyone feels respected in our group. *[Facilitator 1 pulls up Word document from prior session with rules. Facilitator 2 reads prior rules created by the group.]* Now that we’ve read through the rules together, we can get started with our first activity!”

*[If needed or no Word document available, can review expectations with the following:*

*-Do not speak when someone else is speaking.*

*-Don’t leave the room during these sessions unless your parent or family member tells you to do so or there is an emergency.*

*-What’s said in the zoom room stays in the zoom room. It’s okay to talk with your parents or caregivers about what we talk about here.*

*-It’s okay to have different ideas or feelings.*

*-If you feel comfortable, keep your camera on so we can see each other.*

*-Try to join the sessions on time to not miss anything!]*

**Narrative Exercise (22 min):**

3. **12:08-12:30 You and your provider:** Facilitator 1: “Today, our focus will be you and your doctor or medical team. Many of you have to see the doctor regularly to check in and make sure you’re doing okay. Today, we’ll let our inside voices out so they can say how we feel when we see the doctor. You’ll see two photos on the screen here. When you look at these photos, you can choose the one that you like the most. Imagine you’re the teddy bear or the doll in these photos. What does the teddy bear or doll see? What does the teddy bear or doll hear? What does the teddy bear or doll look like? What might the teddy bear or doll say if it doesn’t feel good? What might the teddy bear or doll say if it’s feeling better? You can dance around or make faces, anything you think the little girl or teddy bear may be feeling or want to do. What do you think the teddy bear is feeling? What do you feel when you’re at the doctor?”

*[Facilitator 2 shows photos in PowerPoint while Facilitator 1 is speaking. Facilitator 1 calls on kids to share what they think the teddy bear/doll is hearing/seeing/feeling. Use this as a conversation starter for kids to share what they see/feel when they’re at the doctor as well.]*

*Show photos of Norman Rockwell’s “Doctor and the Doll” and photo of a girl using an otoscope to inspect a teddy bear’s ear.*

**Regulation Break (3 min):**

4. **12:30-12:33PM** **Stretch Break:** Facilitator 2: “Thank you guys for sharing, you all did a wonderful job. You guys have made it to the halfway point, which means it’s time for our stretch break! Everyone can get out of their chair/seated position, stand up and when you’re ready, we’re going to REACH for the sky! Then REACH to the ground. Reach to the LEFT, then reach to the RIGHT. Stand straight up, nice and tall, and time for jumping jacks! Let’s do 10 jumping jacks all together. If you’re not feeling up for jumping jacks today, I invite you to march in place while we count to 10. *[Count from 1 to 10 as the group does jumping jacks]*. And take a breath. You guys have done so well, I want you to take a deep breath, in and out. And when you’re ready, sit back down in your chair/seated position, and we’ll jump right into our next part. *[If anyone needs to use the restroom, it is okay to pause here to allow them to use the restroom and wait to start the next prompt until they rejoin the group if possible.]*

**Narrative Exercise (20 min):**

5. **12:33-12:53: The Two Doctors**: Facilitator 2: “Now that we’ve finished our first half of the session, we’ll move on to get to choose between two options. First, we’ll read a poem together called ‘The Two Doctors,’ and then we’ll spend 5 minutes writing or drawing, depending on which option you choose. If you choose for the poem to be your inspiration, you can spend your 5 minutes writing: What are your do’s and don’ts? How do they make you feel? If the poem doesn’t speak to you and you prefer a different inspiration, you can take the 5 minutes to draw a picture for your doctor, or nurse, or someone you see regularly at the doctor’s office. What do you want them to see that you don’t get to show them during your typical visit? We’ll let you know when you have 1 minute left, and we’ll share what we wrote or drew at the end of the 5 minutes.

*[Facilitator 1 shares the poem from the PowerPoint on the screen while Facilitator 2 is speaking, and notifies the group when there is 1 minute left and when time is up in writing/drawing period.]*

<https://en.miloliza.com/index.php/poems-about-doctors/378-doctors/12096-the-two-doctors>

Who would like to share first?

**Closing (7 min):**

6. **12:53-1PM Grounding Activity**: Facilitator 1: “Thank you all for sharing. At the end of every session, we will close with a short breathing activity. Hopefully this will help you get back to your day’s activities. First, we’ll watch this video to learn how to do the breathing activity together. Then, we’ll each take a few minutes and do the breathing all together.

*[Facilitator 2 pulls up below video and plays it for the group when Facilitator 1 is done speaking:]*

5 finger breathing <https://www.youtube.com/watch?v=HQVZgpyVQ78>

Now that you’ve seen the video, let’s all take a minute and do the exercise together. Take your finger to the base of your thumb, and trace the edge, breathing in, then breath out. Breath in, breath out. *[Continue verbalizing to breath in and out while moving fingers to replicate video.*] When you’re ready, shake your hand, then your arm, then your whole body if you’d like. And when you’ve shaken everything you needed to shake out, come back to stillness, together. Thank you for your time with us today, and we’ll see you in 2 weeks, same time same place. If you think you won’t be able to make it, have your parent or caregiver reach out to Dr. Lanis to discuss next steps. Thanks and have a great rest of the day.

**Session 4: Ages 12-21**

**Session Goal**: To reflect on the interaction between patients and their medical team.

**Gathering and Set Expectations(8 min)**:

1. **12:00-12:05PM Gathering**: Facilitator 1: “Welcome to our third of six sessions where we will get to know each other, share stories, write and create together! For those who may not remember, my name is _____ *[insert your name here]* and I will be one of the facilitators of our group during these sessions. We also have ____ *[insert co-facilitator name here]* as our second facilitator during these sessions. We will be with you throughout this journey and guide everyone along the way.
2. **12:05-12:08 Set Expectations:** Facilitator 2: “Now that we’ve all done introductions, it’s time to remind each other of our rules for these sessions so we all know it’s a safe space to talk with each other. We want to make sure everyone feels respected in our group. *[Facilitator 1 pulls up Word document from prior session with rules. Facilitator 2 reads prior rules created by the group.]* Now that we’ve read through the rules together, we can get started with our first activity!”

*[If needed or no Word document available, can review expectations with the following:*

*-Do not speak when someone else is speaking.*

*-Don’t leave the room during these sessions unless your parent or family member tells you to do so or there is an emergency.*

*-What’s said in the zoom room stays in the zoom room. It’s okay to talk with your parents or caregivers about what we talk about here.*

*-It’s okay to have different ideas or feelings.*

*-If you feel comfortable, keep your camera on so we can see each other.*

*-Try to join the sessions on time to not miss anything!]*

**Narrative Exercise (22 min):**

3. **12:08-12:30 You and your provider:** Facilitator 1: “Today, our focus will be you and your doctor or medical team. Many of you have to see the doctor regularly to check in and make sure you’re doing okay. Today, we’ll write about how we feel when we see the doctor. You’ll see two photos on the screen here. When you look at these photos, you can choose the one that you like the most. Write as if you are one of the characters in either of these paintings. What do you see? What do you hear? What do you feel? What do you wonder? You’ll have 5 minutes to write, and we’ll let you know when you have 1 minute left.”

*[Facilitator 2 shows photos in PowerPoint while Facilitator 1 is speaking. Facilitator 2 keeps time, notifies group when there is 1 minute left and time is up.]*

Who would like to share what they wrote?

*Show photos of J. Bond Francisco’s “The Sick Child” and Norman Rockwell’s “Eye Doctor.”*

**Narrative Exercise (20 min):**

4. **12:30-12:50: The Two Doctors**: Facilitator 2: “Now that we’ve finished our first half of the session, we’ll move on to get to choose between two options. First, we’ll read a poem together called ‘The Two Doctors,’ and then we’ll spend 5 minutes writing or drawing, depending on which option you choose. If you choose for the poem to be your inspiration, you can spend your 5 minutes writing: What are your do’s and don’ts? How do they make you feel? If the poem doesn’t speak to you and you prefer a different inspiration, you can take the 5 minutes to draw a picture for your doctor or a person on your medical team. What do you want them to see that you don’t get to show them during your typical visit? We’ll let you know when you have 1 minute left, and we’ll share what we wrote or drew at the end of the 5 minutes.

*[Facilitator 1 shares the poem from the PowerPoint on the screen while Facilitator 2 is speaking, and notifies the group when there is 1 minute left in writing/drawing period.]*

<https://en.miloliza.com/index.php/poems-about-doctors/378-doctors/12096-the-two-doctors>

Who would like to share first?

**Closing (10 min):**

5. **12:50-1PM Grounding Activity**: Facilitator 1: Thank you all for sharing. Without needing to share with the group, we’re going to write a few questions in the chat for you to think about as you reflect on today’s experience. These questions are also listed on one of the cards you should all have in your packet that we’ll refer back to at the end of each session. You can reflect on these questions while we do our breathing activity to end the session, or if you prefer you can write them down in your notebook after we finish today’s session. We hope these questions will prompt reflections on today’s session. The questions we offer are as follows:

*[Facilitator 2 writes the following in the chat box while Facilitator 1 is speaking. Facilitator 1 pauses for a few seconds between questions to allow for time for the participants to process.]*

I wonder, what did you notice?

I wonder, what was the most important part of today’s group for you?

I wonder, what will you remember tomorrow?

I wonder, what do you wonder about next session?

At the end of every session, we will close with a short breathing activity. You are welcome to ponder the questions we just discussed as we go through this breathing activity, and think to yourself what your answers are. Hopefully this breathing activity will help you get back to your day’s activities. First, we’ll watch this video to learn how to do the breathing activity together. Then, we’ll each take a few minutes and do the breathing all together.

*[Facilitator 2 pulls up below video while Facilitator 1 is speaking:]*

5 finger breathing <https://www.youtube.com/watch?v=HQVZgpyVQ78>

Now that you’ve seen the video, let’s all take a minute and do the exercise together. Take your finger to the base of your thumb, and trace the edge, breathing in, then breath out. Breath in, breath out. *[Continue verbalizing to breath in and out while moving fingers to replicate video.]* When you’re ready, shake your hand, then your arm, then your whole body if you’d like. And when you’ve shaken everything you needed to shake out, come back to stillness, together.

Thank you for your time with us today, and we’ll see you in 2 weeks, same time same place. If you think you won’t be able to make it, have your parent or caregiver reach out to Dr. Lanis to discuss next steps. Thanks and have a great rest of the day!

**Session 5: Ages 6-11**

**Session Goal**: To discuss the experiences of taking medicines regularly and getting lab draws.

**Set Expectations (5 min)**:

1. **12-12:05 Set Expectations:** Facilitator 1: “Welcome back everyone! If your camera is off and you are willing and able to turn the camera on, please do so in order for us to see each other. It’s time to remind each other of our rules for these sessions so we all know it’s a safe space to talk with each other. We want to make sure everyone feels respected in our group. *[Facilitator 1 pulls up Word document from prior session with rules. Facilitator 2 reads prior rules created by the group.]* Now that we’ve read through the rules together, we can get started with our first activity!”

*[If needed or no Word document available, can review expectations with the following:*

*-Do not speak when someone else is speaking.*

*-Don’t leave the room during these sessions unless your parent or family member tells you to do so or there is an emergency.*

*-What’s said in the zoom room stays in the zoom room. It’s okay to talk with your parents or caregivers about what we talk about here.*

*-It’s okay to have different ideas or feelings.*

*-If you feel comfortable, keep your camera on so we can see each other.*

*-Try to join the sessions on time to not miss anything!]*

**Narrative Exercise (15 min):**

2. **12:05-12:20 Everyone’s least favorite: Medicine**: Facilitator 2: “In each of your packets today, you’ll find some very special inspiration, including M&M’s and Swedish Fish! You are allowed to choose one, and we ask that you give the other one to your parent for them to enjoy. For some of you, your parent may have chosen to replace these items with an alternative—in which case we invite you to open the alternative. Once you’ve made your choice, you can open the packet and we invite you to taste one piece. What do you taste? What do you notice? Does it melt in your mouth? Is it sticky? Is it sweet? Do you have more saliva produced as you eat it? Is there a leftover taste in your mouth?

*[Facilitator 2 allows time for group participants to comment on above experience.]*

Now that we’ve had some time to think about what it’s like to eat these items, I’d like you each to think about what it’s like to take your medicines. We’re going to make a Word Document together that shows the words that come to mind when we think about our medicines. What do they taste like? How do you physically feel taking them? How do you emotionally feel when you take your medicines? What side effects do you experience from your medications? How do you deal with these side effects? What does your medication look like when it is working in your body? If you could say anything to your medications, what would you say? You can type your words or answers in the chat box or say them out loud, and we’ll place them in a word document for you all to see.

*[Facilitator 1 shares word document* *and types in responses from participants into the document while sharing the screen for all to see. Examples if need word prompts: bad taste, nauseous, painful, easy, smooth.]*

Thank you for sharing your words. Next, I’d like you each to think about what it’s like to have to get blood draws. We’re going to make a Word Document together that shows the words that come to mind when we think about getting our blood drawn. What does it feel like? What do you feel before the blood is drawn? What do you feel after? What do you feel when the doctor tells you the results? Do you use numbing cream or anything else that helps with the blood draws? You can type your words or answers in the chat box or say them out loud, and we’ll place them in a word document for you all to see.

*[Facilitator 1 shares word document* *and types in responses from participants into the document while sharing the screen for all to see.. Examples if need word prompts: red, vein, poke, needle, doctor, nurse, pain, numbing cream, tourniquet.]*

Now that we’ve put all of the words in the word document, what do you feel seeing the words talking about taking medicines and then about getting your blood drawn?

**Regulation Break (3 min):**

3. **12:20-12:23PM** **Stretch Break:** Facilitator 1: “Thank you all for sharing. You guys have made it to the halfway point, which means it’s time for our stretch break! Everyone can get out of their chair/seated position, stand up and when you’re ready, we’re going to REACH for the sky! Then REACH to the ground. Reach to the LEFT, then reach to the RIGHT. Stand straight up, nice and tall, and time for jumping jacks! Let’s do 10 jumping jacks all together. If you’re not feeling up for jumping jacks today, I invite you to march in place while we count to 10. *[Count from 1 to 10 as the group does jumping jacks]*. And take a breath. You guys have done so well, I want you to take a deep breath, in and out. And when you’re ready, sit back down in your chair/seated position, and we’ll jump right into our next part. *[If anyone needs to use the restroom, it is okay to pause here to allow them to use the restroom and wait to start the next prompt until they rejoin the group if possible.]*

**Narrative Exercise (30 min):**

4. **12:23-12:53: Comics:** Facilitator 1: “For our second activity today, we’re going to create comics! In your packet for this week, you should have papers with boxes on them to create your very own comics. You can choose one of two comics to draw: either about taking medications or about getting your blood drawn.

Now first, let’s walk through what you should think about when making a comic. There are a few important steps to creating a comic:

- First, you want to pick a character. Who is your story about? Is your character a human or a friendly dog? *[Pause and go around, ask each child what character they think they’ll choose.]*
- Then, sketch your character. You can do a few sketches of your character on scratch paper. Draw him/her happy, sad and angry. What does he/she look like running or jumping? *[Pause and ask each chid: what do you think your character would be doing? How would they look?]*
- Next, think about what your character wants? Plan your story by giving your character a hope, want or goal. Then, put something in the way so that your character has to struggle before getting what they want. For example: My character wants _______ but ______ so _____. *[Pause and ask: what would your character want to do? What would make that really hard?]*
- Before you start drawing on your paper with boxes on it, let’s think about how your story will go. Point to each panel and think about what your picture will look like. What will your character be saying in each scene? *[Pause and ask: What would your character do in the pictures?]*
- Finally we will bring your comic to life! In a moment, we will draw pictures on the papers with boxes on them, and add speech bubbles. You can use crayons to add color!

For those who are worried about how to get started with a character, we’ll watch a brief video showing some examples of how to get started. And then, you’ll have 15 minutes to create your masterpieces! At the end, we’ll all come back together to share what we’ve created. If you need more time, that’s okay! You can continue to work on your cartoon after we’re done here today. We’ll let you know when you have 5 minutes left and when you have 1 minute left.

*[Facilitator 2* *pulls up YouTube video of how to draw characters, found here:* [*https://www.youtube.com/watch?v=1Nb_ZDvoWEg*](https://www.youtube.com/watch?v=1Nb_ZDvoWEg) *. This link is also embedded in the shared PowerPoint for the curriculum.]*

As a reminder, you can choose one of two comics to draw: either about taking medications or about getting your blood drawn.

As you start to make your comic, you can think about what does your experience start as? What do you notice when you have to take your medications? What do you notice when you’re getting your blood draws? How do you process the experience? What do you feel physically, what do you feel emotionally? What do you sometimes think about when you’re getting your blood drawn? If you need help, just let us know and we can help you. When you’re ready, you can begin!

*[Participants are given 15 minutes to create their comics. Facilitator 2 notifies the group when they have 5 minutes left, 1 minute left and time is up.]*

Who would like to share what they’ve created?

**Closing (7 min):**

5. **12:53-1PM Grounding Activity**: Facilitator 1: “Thank you all for sharing. At the end of every session, we will close with a short breathing activity. Hopefully this will help you get back to your day’s activities. First, we’ll watch this video to learn how to do the breathing activity together. Then, we’ll each take a few minutes and do the breathing all together.

*[Facilitator 2 pulls up below video and plays it for the group when Facilitator 1 is done speaking:]*

5 finger breathing <https://www.youtube.com/watch?v=HQVZgpyVQ78>

Now that you’ve seen the video, let’s all take a minute and do the exercise together. Take your finger to the base of your thumb, and trace the edge, breathing in, then breath out. Breath in, breath out. *[Continue verbalizing to breath in and out while moving fingers to replicate video.*] When you’re ready, shake your hand, then your arm, then your whole body if you’d like. And when you’ve shaken everything you needed to shake out, come back to stillness, together. Thank you for your time with us today, and we’ll see you in 2 weeks, same time same place. If you think you won’t be able to make it, have your parent or caregiver reach out to Dr. Lanis to discuss next steps. Thanks and have a great rest of the day!

**Session 5: Ages 12-21**

**Session Goal**: To discuss the experiences of taking medicines regularly.

**Set Expectations (5 min)**:

1. **12-12:05 Set Expectations:** Facilitator 1: “Welcome back everyone! If your camera is off and you are willing and able to turn the camera on, please do so in order for us to see each other. It’s time to remind each other of our rules for these sessions so we all know it’s a safe space to talk with each other. We want to make sure everyone feels respected in our group. *[Facilitator 1 pulls up Word document from prior session with rules. Facilitator 2 reads prior rules created by the group.]* Now that we’ve read through the rules together, we can get started with our first activity!”

*[If needed or no Word document available, can review expectations with the following:*

*-Do not speak when someone else is speaking.*

*-Don’t leave the room during these sessions unless your parent or family member tells you to do so or there is an emergency.*

*-What’s said in the zoom room stays in the zoom room. It’s okay to talk with your parents or caregivers about what we talk about here.*

*-It’s okay to have different ideas or feelings.*

*-If you feel comfortable, keep your camera on so we can see each other.*

*-Try to join the sessions on time to not miss anything!]*

**Narrative Exercise (15 min):**

2. **12:05-12:20 Everyone’s least favorite: Medicine**: Facilitator 2: “In each of your packets today, you’ll find some very special inspiration, including M&M’s and Swedish Fish! We ask that you choose one, and that you give the other one to your parent for them to enjoy. For some of you, your parent may have chosen to replace these items with an alternative—in which case we invite you to open the alternative. Once you’ve made your choice, you can open the packet and we invite you to taste one piece. What do you taste? What do you notice? Does it melt in your mouth? Do you have more saliva produced as you eat it? Is there a leftover taste in your mouth?

*[Facilitator 2 allows time for group participants to comment on above experience.]*

Now that we’ve had some time to think about what it’s like to eat these items, I’d like you each to think about what it’s like to take your medicines. We’re going to make a Word Document together that shows the words that come to mind when we think about our medicines. What do they taste like? How do you physically feel taking them? How do you emotionally feel when you take your medicines? What side effects do you experience from your medications? How do you deal with these side effects? What does your medication look like when it is working in your body? If you could say anything to your medications, what would you say? You can type your words or answers in the chat box or say them out loud, and we’ll place them in a word document for you all to see.

*[Facilitator 1 shares word document* *and types in responses from participants into the document while sharing the screen for all to see. Examples if need word prompts: bad taste, nauseous, painful, easy, smooth.]*

Thank you for sharing your words. Next, I’d like you each to think about what it’s like to have to get blood draws. We’re going to make a Word Document together that shows the words that come to mind when we think about getting our blood drawn. What does it feel like? What do you feel before the blood is drawn? What do you feel after? What do you feel when the doctor tells you the results? Do you use numbing cream or anything else that helps with the blood draws? You can type your words or answers in the chat box or say them out loud, and we’ll place them in a word document for you all to see.

*[Facilitator 1 shares word document* *and types in responses from participants into the document while sharing the screen for all to see. Examples if need word prompts: red, vein, poke, needle, doctor, nurse, pain, numbing cream, tourniquet.]*

Now that we’ve put all of the words in the word document, what do you feel seeing the words talking about taking medicines and then about getting your blood drawn?

**Narrative Exercise (30 min):**

3. **12:20-12:50: Comics:** Facilitator 1: “For our second activity today, we’re going to create comics! In your packet for this week, you should have papers with boxes on them to create your very own comics. You can choose one of two comics to draw: either about taking medications or about getting your blood drawn.

Now first, let’s walk through what you should think about when making a comic. There are a few important steps to creating a comic:

- First, you want to pick a character. Who is your story about? Is your character a human or a friendly dog?
- Then, sketch your character. You can do a few sketches of your character on scratch paper. Draw him/her happy, sad and angry. What does he/she look like running or jumping?
- Next, think of a plot. What does your character want? Plan your story by giving your character a hope, want or goal. Then, put something in the way so that your character has to struggle before getting what they want. For example: My character wants _______ but ______ so _____.
- Before you start drawing on your panels (the paper with boxes on it), plan how your story will go. Point to each panel and plan what your picture will look like. What will your character be saying in each scene?
- Finally bring your comic to life! Draw pictures on the papers with boxes on them, and add speech bubbles. Use crayons to add color!

For those who are worried about how to get started with a character, we’ll watch a brief video showing some examples of how to get started. And then, you’ll have 15 minutes to create your masterpieces! At the end, we’ll all come back together to share what we’ve created. If you need more time, that’s okay! You can continue to work on your cartoon after we’re done here today. We’ll let you know when you have 5 minutes left and when you have 1 minute left.

As a reminder, you can choose one of two comics to draw: either about taking medications or about getting your blood drawn. As you start to make your comic, you can think about what does your experience start as? What do you notice when you have to take your medications? What do you notice when you’re getting your blood draws? How do you process the experience? What do you feel physically, what do you feel emotionally? What do you sometimes think about when you’re getting your blood drawn? If you need help, just let us know and we can help you. When you’re ready, you can begin!

*[Facilitator 2* *pulls up YouTube video of how to draw characters, found here:* [*https://www.youtube.com/watch?v=1Nb_ZDvoWEg*](https://www.youtube.com/watch?v=1Nb_ZDvoWEg) *. This link is also embedded in the shared PowerPoint for the curriculum. This video is played, and then participants are given 15 minutes to create their comics. Facilitator 2 notifies the group when they have 5 minutes left, 1 minute left, and time is up.]*

Who would like to share what they’ve created?

**Closing (10 min):**

4. **12:50-1PM Grounding Activity**: Facilitator 1: Thank you all for sharing. Without needing to share with the group, we’re going to write a few questions in the chat for you to think about as you reflect on today’s experience. These questions are also listed on one of the cards you should all have in your packet that we’ll refer back to at the end of each session. You can reflect on these questions while we do our breathing activity to end the session, or if you prefer you can write them down in your notebook after we finish today’s session. We hope these questions will prompt reflections on today’s session. The questions we offer are as follows:

*[Facilitator 2 writes the following in the chat box while Facilitator 1 is speaking. Facilitator 1 pauses for a few seconds between questions to allow for time for the participants to process.]*

I wonder, what did you notice?

I wonder, what was the most important part of today’s group for you?

I wonder, what will you remember tomorrow?

I wonder, what do you wonder about next session?

At the end of every session, we will close with a short breathing activity. You are welcome to ponder the questions we just discussed as we go through this breathing activity, and think to yourself what your answers are. Hopefully this breathing activity will help you get back to your day’s activities. First, we’ll watch this video to learn how to do the breathing activity together. Then, we’ll each take a few minutes and do the breathing all together.

*[Facilitator 2 pulls up below video while Facilitator 1 is speaking:]*

5 finger breathing <https://www.youtube.com/watch?v=HQVZgpyVQ78>

Now that you’ve seen the video, let’s all take a minute and do the exercise together. Take your finger to the base of your thumb, and trace the edge, breathing in, then breath out. Breath in, breath out. *[Continue verbalizing to breath in and out while moving fingers to replicate video.]* When you’re ready, shake your hand, then your arm, then your whole body if you’d like. And when you’ve shaken everything you needed to shake out, come back to stillness, together.

a. Thank you for your time with us today, and we’ll see you in 2 weeks, same time same place. If you think you won’t be able to make it, have your parent or caregiver reach out to Dr. Lanis to discuss next steps. Thanks and have a great rest of the day!I wonder, has your color changed from the beginning?

**Session 6: Ages 6-11**

**Session Goal**: To manage expectations, emphasize the power of hope, and reflect on the experience.

**Set Expectations (3 min)**:

1. **12-12:03 Set Expectations:** Facilitator 1: “Welcome back everyone! If your camera is off and you are willing and able to turn the camera on, please do so in order for us to see each other. It’s time to remind each other of our rules for these sessions so we all know it’s a safe space to talk with each other. We want to make sure everyone feels respected in our group. *[Facilitator 1 pulls up Word document from prior session with rules. Facilitator 2 reads prior rules created by the group.]* Now that we’ve read through the rules together, we can get started with our first activity!”

*[If needed or no Word document available, can review expectations with the following:*

*-Do not speak when someone else is speaking.*

*-Don’t leave the room during these sessions unless your parent or family member tells you to do so or there is an emergency.*

*-What’s said in the zoom room stays in the zoom room. It’s okay to talk with your parents or caregivers about what we talk about here.*

*-It’s okay to have different ideas or feelings.*

*-If you feel comfortable, keep your camera on so we can see each other.*

*-Try to join the sessions on time to not miss anything!]*

**Sharing (5 min):**

2. **12:03-12:08 Reviewing Comics**: Facilitator 1: “Before we start with our activities today, we wanted to open up the floor to share updated comics from our last session! If you have the comic with you you’re welcome to show the comic in your camera for all of us to see! If you don’t have the comic but would like to share any changes since the last session, please share what you chose to include in your comic.”

**Narrative Exercise (22 min):**

3. **12:08-12:30 Resilience**: Facilitator 2: “Today, we’ll start our day with a poem read by Serena Williams, a former professional tennis player thought to be one of the greatest tennis players of all time. She reads a poem entitled “Still I Rise” by Maya Angelou, a poet and civil rights activist. After we finish hearing the poem, we’ll take 5 minutes to write: What does this poem make you feel? Write what helps you stand up when you’re having a tough day. We’ll let you know when we have 1 minute left in the writing period, and when we’re finished, we’ll share our writings.

*[Facilitator 1 pulls up the Still I Rise Video, found here* <https://www.youtube.com/watch?v=TZO08C5vL2A> *, also embedded in the shared PowerPoint. Facilitator 1 notifies group where there is 1 minute left and time is up.]*

Who would like to share what they wrote?

**Regulation Break (3 min):**

4. **12:30-12:33PM** **Stretch Break:** Facilitator 2: “You guys have made it to the halfway point, which means it’s time for our stretch break! Everyone can get out of their chair/seated position, stand up and when you’re ready, we’re going to REACH for the sky! Then REACH to the ground. Reach to the LEFT, then reach to the RIGHT. Stand straight up, nice and tall, and time for jumping jacks! Let’s do 10 jumping jacks all together. If you’re not feeling up for jumping jacks today, I invite you to march in place while we count to 10. *[Count from 1 to 10 as the group does jumping jacks]*. And take a breath. You guys have done so well, I want you to take a deep breath, in and out. And when you’re ready, sit back down in your chair/seated position, and we’ll jump right into our next part. *[If anyone needs to use the restroom, it is okay to pause here to allow them to use the restroom and wait to start the next prompt until they rejoin the group if possible.]*

**Reflections/Feedback (17 min):**

5. **12:33-12:50 Reflections:** Facilitator 2: “As we come to a close with our last session of the series, we wanted to offer a space to reflect on this experience together. As you reflect on the sessions we’ve had can you share one thing that was important to you and why? One thing we can do better in the future, and one thing we should just get rid of?”

*[Facilitator 1 takes notes while Facilitator 2 guides discussion around pros/cons of the experiences, preferences and ideas of things that could/should change.]*

Thank you all for your feedback! If you liked this, tell your parents and they can let us know! In your final ‘Week 6’ envelope this week, you’ll find a postcard. We invite you to write words or draw a picture for yourself in this space after our session today, that you can keep as a memory from this time we’ve spent together. There are many other ways to get involved with poetry writing, including Pongo Poetry Project with kids. You can even submit poetry and writings to them and win prizes! We’ve included these resources and more in your packets that you and your caregivers can look through if you’re interested in learning more.

**Closing (7 min):**

6. **12:53-1PM Grounding Activity**: Facilitator 1: “Thank you all for sharing. At the end of every session, we will close with a short breathing activity. Hopefully this will help you get back to your day’s activities. First, we’ll watch this video to learn how to do the breathing activity together. Then, we’ll each take a few minutes and do the breathing all together.

*[Facilitator 2 pulls up below video and plays it for the group when Facilitator 1 is done speaking:]*

5 finger breathing <https://www.youtube.com/watch?v=HQVZgpyVQ78>

Now that you’ve seen the video, let’s all take a minute and do the exercise together. Take your finger to the base of your thumb, and trace the edge, breathing in, then breath out. Breath in, breath out. *[Continue verbalizing to breath in and out while moving fingers to replicate video.*] When you’re ready, shake your hand, then your arm, then your whole body if you’d like. And when you’ve shaken everything you needed to shake out, come back to stillness, together. Thank you for your time with us today, and for spending time with us over the past 3 months. Thanks and have a great rest of the day!

**Session 6: Ages 12-21**

**Session Goal**: To manage expectations, emphasize the power of hope, and reflect on the experience.

**Set Expectations (3 min)**:

1. **12-12:03 Set Expectations:** Facilitator 1: “Welcome back everyone! If your camera is off and you are willing and able to turn the camera on, please do so in order for us to see each other. It’s time to remind each other of our rules for these sessions so we all know it’s a safe space to talk with each other. We want to make sure everyone feels respected in our group. *[Facilitator 1 pulls up Word document from prior session with rules. Facilitator 2 reads prior rules created by the group.]* Now that we’ve read through the rules together, we can get started with our first activity!”

*[If needed or no Word document available, can review expectations with the following:*

*-Do not speak when someone else is speaking.*

*-Don’t leave the room during these sessions unless your parent or family member tells you to do so or there is an emergency.*

*-What’s said in the zoom room stays in the zoom room. It’s okay to talk with your parents or caregivers about what we talk about here.*

*-It’s okay to have different ideas or feelings.*

*-If you feel comfortable, keep your camera on so we can see each other.*

*-Try to join the sessions on time to not miss anything!]*

**Sharing (5 min):**

2. **12:03-12:08 Reviewing Comics**: Facilitator 1: “Before we start with our activities today, we wanted to open up the floor to share updated comics from our last session! If you have the comic with you you’re welcome to show the comic in your camera for all of us to see! If you don’t have the comic but would like to share any changes since the last session, please share what you chose to include in your comic.”

**Narrative Exercise (22 min):**

3. **12:08-12:30 Resilience**: Facilitator 2: “Today, we’ll start our day with a poem read by Serena Williams, a former professional tennis player thought to be one of the greatest tennis players of all time. She reads a poem entitled “Still I Rise” by Maya Angelou, a poet and civil rights activist. After we finish hearing the poem, we’ll take 5 minutes to write: What does this poem make you feel? Write what helps you stand up when you’re having a tough day. We’ll let you know when we have 1 minute left in the writing period, and when we’re finished, we’ll share our writings.

*[Facilitator 1 pulls up the Still I Rise Video, found here* <https://www.youtube.com/watch?v=TZO08C5vL2A> *, also embedded in the shared PowerPoint. Facilitator 1 notifies group where there is 1 minute left and time is up.]*

Who would like to share what they wrote?

**Reflections/Feedback (20 min):**

4. **12:30-12:50 Reflections:** Facilitator 2: “As we come to a close with our last session of the series, we wanted to offer a space to reflect on this experience together. As you reflect on the sessions we’ve had can you share one thing that was important to you and why? One thing we can do better in the future, and one thing we should just get rid of?”

*[Facilitator 1 takes notes while Facilitator 2 guides discussion around pros/cons of the experiences, preferences and ideas of things that could/should change.]*

Thank you all for your feedback! If you liked this, tell your parents and they can let us know! In your final ‘Week 6’ envelope this week, you’ll find a postcard. We invite you to write words or draw a picture for yourself in this space after our session today, that you can keep as a memory from this time we’ve spent together. There are many other ways to get involved with poetry writing, including Pongo Poetry Project with kids. You can even submit poetry and writings to them and win prizes! We’ve included these resources and more in your packets that you and your caregivers can look through if you’re interested in learning more.

**Closing (10 min):**

5. **12:50-1PM Grounding Activity**: Facilitator 1: Thank you all for sharing. Without needing to share with the group, we’re going to write a few questions in the chat for you to think about as you reflect on today’s experience. These questions are also listed on one of the cards you should all have in your packet that we’ll refer back to at the end of each session. You can reflect on these questions while we do our breathing activity to end the session, or if you prefer you can write them down in your notebook after we finish today’s session. We hope these questions will prompt reflections on today’s session. The questions we offer are as follows:

*[Facilitator 2 writes the following in the chat box while Facilitator 1 is speaking. Facilitator 1 pauses for a few seconds between questions to allow for time for the participants to process.]*

I wonder, what did you notice?

I wonder, what was the most important part of today’s group for you?

I wonder, what will you remember tomorrow?

I wonder, what do you wonder about next session?

At the end of every session, we will close with a short breathing activity. You are welcome to ponder the questions we just discussed as we go through this breathing activity, and think to yourself what your answers are. Hopefully this breathing activity will help you get back to your day’s activities. First, we’ll watch this video to learn how to do the breathing activity together. Then, we’ll each take a few minutes and do the breathing all together.

*[Facilitator 2 pulls up below video while Facilitator 1 is speaking:]*

5 finger breathing <https://www.youtube.com/watch?v=HQVZgpyVQ78>

Now that you’ve seen the video, let’s all take a minute and do the exercise together. Take your finger to the base of your thumb, and trace the edge, breathing in, then breath out. Breath in, breath out. *[Continue verbalizing to breath in and out while moving fingers to replicate video.]* When you’re ready, shake your hand, then your arm, then your whole body if you’d like. And when you’ve shaken everything you needed to shake out, come back to stillness, together.

Thank you for your time with us today, and for spending time with us over the past 3 months. Thanks and have a great rest of the day!
